# Hybridization Dynamics and Extensive Introgression in the *Daphnia longispina* Species Complex: New Insights from a High-Quality *Daphnia galeata* Reference Genome

**DOI:** 10.1093/gbe/evab267

**Published:** 2021-12-02

**Authors:** Jana Nickel, Tilman Schell, Tania Holtzem, Anne Thielsch, Stuart R Dennis, Birgit C Schlick-Steiner, Florian M Steiner, Markus Möst, Markus Pfenninger, Klaus Schwenk, Mathilde Cordellier

**Affiliations:** 1 Institute of Zoology, Universität Hamburg, Germany; 2 LOEWE Centre for Translational Biodiversity Genomics (LOEWE-TBG), Frankfurt am Main, Germany; 3 Department of Ecology, University of Innsbruck, Austria; 4 Molecular Ecology, Institute for Environmental Sciences, University Koblenz-Landau, Landau in der Pfalz, Germany; 5 Department of Aquatic Ecology, EAWAG, Dübendorf, Switzerland; 6 Molecular Ecology, Senckenberg Biodiversity and Climate Research Centre, Frankfurt, Germany; 7 IoME, Gutenberg University, Mainz, Germany

**Keywords:** introgression, hybridization, resting eggs, species complex, whole-genome amplification, genome assembly

## Abstract

Hybridization and introgression are recognized as an important source of variation that influence adaptive processes; both phenomena are frequent in the genus *Daphnia*, a keystone zooplankton taxon in freshwater ecosystems that comprises several species complexes. To investigate genome-wide consequences of introgression between species, we provide here the first high-quality genome assembly for a member of the *Daphnia longispina* species complex, *Daphnia galeata*. We further resequenced 49 whole genomes of three species of the complex and their interspecific hybrids both from genotypes sampled in the water column and from single resting eggs extracted from sediment cores. Populations from habitats with diverse ecological conditions offered an opportunity to study the dynamics of hybridization linked to ecological changes and revealed a high prevalence of hybrids. Using phylogenetic and population genomic approaches, we provide first insights into the intra- and interspecific genome-wide variability in this species complex and identify regions of high divergence. Finally, we assess the length of ancestry tracts in hybrids to characterize introgression patterns across the genome. Our analyses uncover a complex history of hybridization and introgression reflecting multiple generations of hybridization and backcrossing in the *Daphnia longispina* species complex. Overall, this study and the new resources presented here pave the way for a better understanding of ancient and contemporary gene flow in the species complex and facilitate future studies on resting egg banks accumulating in lake sediment.

Significance

Hybridization and introgression frequently occur in the water flea genus *Daphnia*. Until now, genomic resources to investigate the amount of introgression in the *Daphnia longispina* species complex, a widespread taxon in European lakes and keystone grazer of phytoplankton, were limited. We provide here a genome assembly and the first genome-wide analysis of several species of the complex, based on samples from the water column and resting eggs from sediment cores. Using phylogenetic and population genomic approaches, we measure intra- and interspecific genome-wide variability and identify regions of high divergence. Our study reveals extensive gene flow between taxa, and later generation hybrids originating from several rounds of backcrossing. It paves the way for further studies aiming at understanding how species boundaries are maintained in the face of gene flow.

## Introduction

Gene flow between species can be pervasive and can affect substantial parts of the genome. Hybridization and introgression are recognized as an important source of variation that can influence adaptive processes in plants, animals, yeast, and fungi (reviewed in Abbott et al. [2013]; Arnold and Martin [2009]). The amount of realized gene flow varies among taxa and along the genome; it is governed by intrinsic genomic features such as recombination rate, structural variation, and intrinsic incompatibilities, as well as the species’ biology and ecology including ecological and sexual selection, migration, and mode of reproduction.

How can species in diversifying clades frequently hybridize and show introgression but nevertheless maintain species boundaries? A growing body of literature provides examples for a high variety of systems where speciation occurs in the face of gene flow (Fraïsse et al. 2014; Meier et al. 2017; Martin et al. 2019). However, it is important to recognize that these systems are distributed along a wide spectrum. On one side of this spectrum, hybridization occurs but is not followed by introgression for several reasons such as reduced hybrid fertility or strong selection against hybrid phenotypes, leading to rapid hybrid breakdown. Barth et al. (2020) found that species boundaries in tropical eels are stable despite millions of years of hybridization, and also observed very few admixed individuals beyond F1 and first-generation backcrosses. The hybrid breakdown observed in this system reduces the likelihood of introgression via backcrossing. On the other side of the spectrum, hybridization is followed by introgression, and ongoing exchange of genetic information between species (Martin et al. 2013; Butlin et al. 2014; Doellman et al. 2018; Kaiser et al. 2021). Several empirical studies (Canestrelli et al. 2017; Schreiber and Pfenninger 2021) as well as theoretical models (Yeaman and Whitlock 2011; Flaxman et al. 2014; Rafajlović et al. 2016) suggest the possibility of intermediate constant equilibrium states, meaning that certain parts of the genome remain diverged (islands or continents of divergence), whereas others are freely exchanged among closely related species without ever reaching complete genomic isolation.

Recurrent hybridization and introgression are frequent in the genus *Daphnia* (Crustacea, Cladocera) Members of the genus have served as ecological model organisms for over a century (Miner et al. 2012), and the first crustacean genome to be sequenced was that of a member of the *Daphnia pulex* species complex (Colbourne et al. 2011). Since then, the genomes of 45 crustaceans have been sequenced with a focus on species of economic or medical interest (NCBI, last accessed January 2021). Despite their key role in marine and freshwater food webs around the globe, genomic resources for zooplanktonic species are still scarce. In many aquatic food webs, zooplanktonic crustaceans link primary production by phytoplankton and secondary consumers, such as planktivorous fish and larger invertebrate species (Gannon and Stemberger 1978; Gliwicz 1990; Lampert and Sommer 2007).

*Daphnia* are highly phenotypically plastic and a textbook example for inducible defense mechanisms (Tollrian and Harvell 1999), as they respond to variation in predation risk through spectacular changes in morphology. Further, *Daphnia* are cyclical parthenogens and hence able to alternate between asexual and sexual reproduction. They reproduce asexually through longer periods of time, and the product of sexual reproduction events (usually seasonal) are resting eggs able to withstand adverse conditions for decades and even centuries (Frisch et al. 2014). Resting eggs extracted from sediment cores can be hatched, and ancient genotypes brought to life (reviewed in Orsini et al. 2013]). Moreover, the DNA preserved in those resting eggs can be directly analyzed with various molecular methods (Cousyn et al. 2001; Lack et al. 2018; Dziuba et al. 2020). Thus, cyclical parthenogenesis, biological archives in lake sediments and high levels of phenotypic plasticity make *Daphnia* a particularly interesting model for evolutionary studies.

The genus *Daphnia* is composed of two subgenera, *Ctenodaphnia* and *Daphnia*, and two groups are delimited within the subgenus *Daphnia*: the *D. pulex* group sensu lato and the *Daphnia longispina* group sensu lato (see Adamowicz et al. [2009]). The latter is sometimes also referred to as subgenus *Hyalodaphnia* and includes the *D. longispina* species complex (DLSC) (Petrusek, Hobæk, et al. 2008). The two *Daphnia* groups are highly differentiated and share their most recent common ancestor around 30 Ma (MRCA *D. longispina–D. pulex* group, MRCA *D. longispina–D. pulex* group, Cornetti et al. 2019). Members of the genus *Daphnia* show little variation in chromosome number, with most species having ten pairs of chromosomes, except for the *D. pulex* group with *n* = 12 (Trentini 1980; Beaton and Hebert 2004). All sequenced and assembled *Daphnia* genomes so far belong either to the *D. pulex* group or the subgenus *Ctenodaphnia*, however no high-quality reference genome of the third major group, the *D. longispina* group (*Hyalodaphnia*) is published.

The prevalence of hybridization in the genus *Daphnia* across taxa and ecosystems and its impact on their evolutionary history has intrigued researchers for decades (Wolf 1987; Schwenk 1993; Vergilino et al. 2011). In contrast to many other well-studied hybrid systems (Barton and Hewitt 1985) with clear defined hybrid zones where species’ ranges overlap, the distribution of *Daphnia* species and their hybrids is more of a fragmented nature: they occupy lake and pond ecosystems that vary in their ecological characteristics and hence constitute a mosaic across the landscape. Ecologically differentiated taxa and their hybrids are thus distributed across habitat patches (Harrison 1986). Within these patches, the possibility to interrogate biological archives also revealed fluctuations in *Daphnia* community composition over time (Brede et al. 2009; Alric et al. 2016), associated with hybridization events among species in some cases. Variation in hybridization events across time and among habitats has often been observed in correlation with ecological changes, such as eutrophication or global change (Keller et al. 2008; Brede et al. 2009; Rellstab et al. 2011; Spaak et al. 2012; Dziuba et al. 2020; Cordellier et al. 2021).

Members of the DLSC inhabit many large ponds and lakes in central and northern Europe, and three of them have been particularly well studied: *Daphnia galeata*, *D. longispina*, and *Daphnia cucullata* (Petrusek, Hobæk, et al. 2008). These species can coexist, but earlier studies suggest gene flow among them is limited (Spaak 2004). Despite their obviously ancient divergence (Schwenk et al. 2000), DLSC species are still able to form interspecific hybrids, although not all combinations are equally likely to lead to viable and fertile individuals (Schwenk et al. 2001). A mechanism preventing gene flow among species might be their different ecological preferences, for example, regarding trophic level (Spaak et al. 2012), food quality (Seidendorf et al. 2007), and predation pressure (Spaak and Hoekstra 1997; Petrusek, Seda, et al. 2008).

Up to now, genetic markers available to study hybridization in the DLSC are limited to allozymes (Wolf and Mort 1986), a few mitochondrial regions (Schwenk 1993), a dozen microsatellite markers (Brede et al. 2006; Thielsch et al. 2012) and a few further nuclear loci (Billiones et al. 2004; Skage et al. 2007; Rusek et al. 2015). Seminal studies such as Brede et al. (2009) and Limburg and Weider (2002) first made use of microsatellite markers to analyze environmentally driven shifts in allelic frequencies, species, and hybrid composition of the DLSC communities in Lake Constance and Belauer See over time, respectively. Further, a number of studies addressed the spatial distribution of DLSC species/taxa with these markers (Griebel et al. 2016; Thielsch et al. 2017; Ma et al. 2019). These low-resolution markers allowed to identify hybrid individuals and brought evidence for introgression but could not provide the resolution necessary to either assess how pervasive introgression is or how it varies across the genome. Further, it is not clear whether introgression occurs among all three species to the same extent. Given the ubiquitous hybridization among the DLSC taxa, the question also arises why they are still well distinguishable species. Whether the DLSC represents a case of incipient speciation, introgression after secondary contact, speciation reversal, or has reached an intermediate constant equilibrium state, among other possibilities, can only be answered with genome-wide analyses empowered by a high-quality genome assembly.

Here, we present a high-quality assembly for *D. galeata*, thus filling an important gap for *Daphnia* whole-genome studies. Furthermore, to facilitate genome-wide assessments of divergence across species and of introgression between species, we conducted genome-wide resequencing studies in the DLSC. We analyzed whole-genome sequences of parental species and their interspecific hybrids, both from genotypes obtained in the wild and maintained in laboratories, and from single resting eggs extracted from sediment cores. We provide first insights into the intra- and interspecific genome-wide variability in this species complex and identify regions of high divergence. We reconstructed the phylogenetic relationships in the species complex using whole mitochondrial genomes. Finally, we assess the length of ancestry tracts in different classes of hybrids to characterize introgression patterns. Our study paves the way for long-awaited analyses on the dynamics of introgression in this complex and exploitation of the unique opportunity this group has to offer: a window of more than one hundred years of evolution in action.

## Results

### Genome Assembly

The raw assembly was obtained by combining PacBio long reads (1,679,290, 11.52 Gb) and Illumina short reads (70,310,338, 9.79 Gb after trimming) and using the hybrid assembler RA (https://github.com/lbcb-sci/ra, last accessed January 2021). It originally comprised 1,415 contig sequences covering a total length of 153.6 Megabases (Mb), with an N50 value of 172 kilobases (kb) and a slightly elevated GC content (40.02% supplementary table S3, Supplementary Material online) compared with the values expected for a *Daphnia* species (see table 1). According to an analysis based on coverage and GC content of the contig sequences conducted with blobtools (Laetsch and Blaxter 2017), a portion of the assembly consisted of non-*Daphnia* contigs, which could then be removed (267 contigs, equaling 22.97 Mb). Consequently, GC content decreased to 38.75%, nearing the values obtained for other *Daphnia* assemblies (see table 1 for an overview). The application of this filter as well as the exclusion of the mitochondrial genome led to a decrease in the number of sequences and the total length of the assembly. Iterative scaffolding led to a decrease in the total number of sequences. This together with a substantial increase in N50 resulted in a highly contiguous assembly, with a total length of 133,304,63 bp, an N50 of 756.7 kb and only 346 sequences, that is, on an average 30 sequences per chromosome. Contiguity statistics for the different assembling steps are given in supplementary table S3, Supplementary Material online.

**Table 1:** Assembly Metrics and Annotation Statistics for the Present Assembly and Two Previously Published *Daphnia* Assemblies

Species	*D. galeata*	*D. pulex* (Ye et al. 2017)	*D. magna* (Lee et al. 2019)
Strain	M5	PA42	SK
Assembly metrics			
No. scaffolds	346	493	4,192
Largest scaffold (bp)	2,950,711	7,584,612	16,359,456
Total length (bp)	133,304,630	189,550,516	122,937,721
N50 (bp)	756,671	1,160,003	10,124,675
L50 (bp)	48	36	5
GC (%)	38.75	40.39	40.54
No. *N*’s	120,845	4,006,006	82,97,703
No. *N*’s per 100 kb	90.65	2,113.42	6,749.52
Annotation			
Number			
Gene	15,845	18,440	15,721
mRNA	16,774	18,440	15,721
Exon	117,364	128,688	95,203
CDS	119,402	118,916	94,047
Mean			
mRNAs/gene	1.06	1	1
Exons/mRNA	7.00	6.98	6.06
CDSs/mRNA	7.12	6.45	5.98
Median length (bp)			
Gene	2,097	1,919.5	1,586
mRNA	2,142	1,919.5	1,521
Exon	167	162	160
Intron	74		
CDS	152	144	159
Total space (bp)			
Gene	51,689,473	53,936,938	37,505,261
mRNA	51,689,329	53,936,938	36,178,687
Exon	29,314,592	30,208,483	22,336,755
CDS	25,132,876	23,586,918	21,881,778
Single			
Exon mRNA	663	144	1,775
CDS mRNA	710	0	0
BUSCO *N* = 1,066			
C	94.3	94.1	97.0
S	91.7	82.6	95.3
D	2.6	11.5	1.7
F	0.7	3.5	1.7
M	5.0	2.4	1.3
DOGMA *N* = 4,222	93.63	91.43	93.91

Note.—Contiguity statistics of the annotation were calculated excluding tRNAscan results. BUSCO 3.0.2 was executed in protein mode for the different MAKER rounds. Conserved domain arrangements were searched with Pfam scan 1.6 and DOGMA 3.4. Results for BUSCO and DOGMA completeness statistics are given in percent.

Mapping the filtered Illumina reads with bwa mem (Li 2013) and PacBio reads with Minimap 2.17 (Li 2018), resulted in a mapping rate of, respectively, 94.1% and 85.5%. The coverage distribution can be seen in supplementary figure S1*B*, Supplementary Material online.

According to blobtools results, no contamination could be identified in the final assembly (supplementary fig. S1*A*, Supplementary Material online). Remaining scaffolds (12, amounting to a total length 1.79 Mb) with taxonomic assignment other than Arthropoda were kept because coverage and GC are similar to *D. galeata* scaffolds and taxonomic assignment alone might be false positive. Further, the completeness assessment through BUSCO (Simão et al. 2015, Arthropoda set, odb9) indicated 95.7% of complete single copy core orthologs and a very low duplication rate (C: 95.7% [S: 94.7%, D: 1.0%], F: 0.8%, M: 3.5%, n: 1,066). The genome size was estimated based on mapped nucleotides and mode of the coverage distribution by backmap 0.3 (https://github.com/schellt/backmap, last accessed January 2021), resulting in 156.86 and 178.03 Mb for Illumina (52*×*) and PacBio (26*×*) respectively, and by k-mer based approach using GenomeScope resulting in a size of 150.6 Mb.

When compared with other published full genomes for *Daphnia* species, the *D. galeata* final assembly is shorter than both *D. pulex* assemblies (Colbourne et al. 2011; Ye et al. 2017), and roughly the same size as *Daphnia magna* (Lee et al. 2019), which also has ten chromosomes (table 1). The GC content is lower, which can be attributed to the strict filtering for contamination applied pre- and postassembly, a procedure not applied in the other *Daphnia* assemblies, to our knowledge. Even though Lee et al. (2019) and Ye et al. (2017) treated the animals with antibotics before sequencing this suggests that these genome assemblies contain more contigs of bacterial origin than the *D. galeata* assembly. Thanks to the use of long-read data, iterative scaffolding and gap filling; the number and length of assembly gaps (Ns) are substantially lower and contiguity is high (but see supplementary table S3, Supplementary Material online).

### Genome Annotation

After applying RepeatMasker (Smit et al. 2013–2015) with the custom repeat library described in the Materials and Methods section, 21.9% of the assembly was masked. The distribution of masked fraction per repeat element can be found in supplementary table S5, Supplementary Material online.

The final annotation with MAKER (Holt and Yandell 2011) predicts 15,845 genes with a median length of 2,097 base pairs. There is an average of 1.06 mRNAs per gene and seven exons per mRNA (table 1). The total number of predicted mRNA substantially differs from the number of transcripts previously published for this species (32,903, Huylmans et al. 2016). This is not surprising, as this transcriptome assembly did not make use of protein evidence we included here, and might contain isoforms. Further, it was based on a pool of mRNA from different clonal lines, and the assembly process might have been impeded by allelic diversity. As further quality criterion, the Annotation Editing Distance (AED) was compared across the three MAKER rounds and is visualized in supplementary figure S4, Supplementary Material online. AED improved mostly between rounds 1 and 2 of the annotation but only marginally with a further round.

A high percentage of protein sequences could be annotated: 15,898 (94.78%) with InterProScan (Jones et al. 2014) and 15,960 (95.15%) with BLAST against Swiss-Prot. With this combination of searches, a hit within InterProScan and BLAST was found for 16,675 protein sequences (99.41%). GeneOntology annotation was possible for 9,555 sequences (56.96%). A detailed overview of the functional annotated sequences per database or search algorithm is shown in supplementary table S7, Supplementary Material online.

### Genotyping

**Fig. 1.**
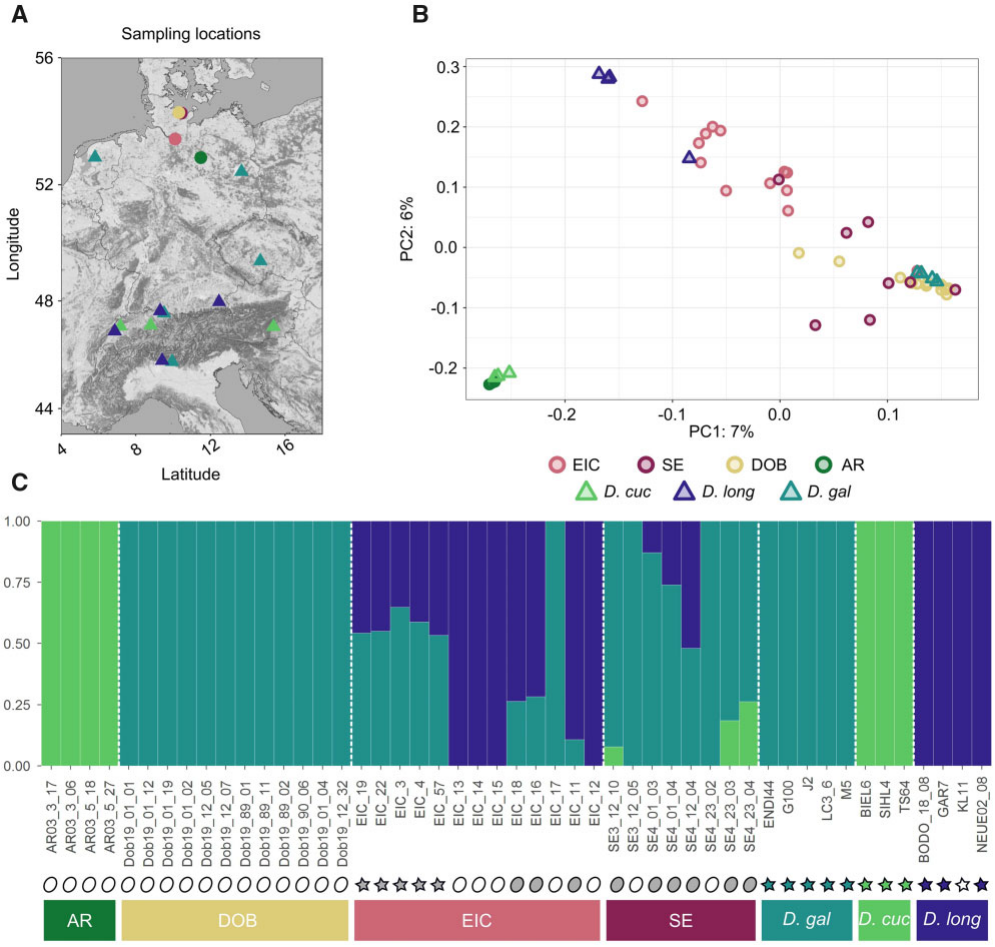
Parental species: *Daphnia gal*: *D. galeata*, *D. long*: *D. longispina*, *D. cuc*; *D. cucullata*, populations: AR, Arendsee; Dob, Dobersdorfer See; SE, Selenter See; EIC, Eichbaumsee. Color and symbol coding are consistent throughout panels (*A*) and (*B*). (*A*) Map of the sampling locations. (*B*) PCA plot obtained with SNPrelate, including loci with linkage *r*^2^ <0.5 within 500-kb sliding windows. (*C*) Admixture plot obtained with *K* = 3. Symbols indicate the sample type: oval for genotypes sequenced directly from resting eggs, stars for genotypes sampled in the water column and propagated clonally in the laboratory prior to sequencing. Symbol filling indicates how these genotypes were classified in subsequent analyses: white for nonadmixed genotypes; grey for admixed genotypes; and green, blue, and teal for genotypes used as representatives for parental species. Bottom bars are color coded to match the color scheme used in panels (*A*) and (*B*).

Short-read sequence data were generated for 72 individuals: 17 unamplified DNA samples from isofemale clonal lines and 55 whole-genome amplification (WGA) samples (conducted on single resting eggs) that passed PCR contamination checks. After screening for contamination and removing data sets with only very few reads mapping to the *D. galeata* reference, 49 single genotypes remained: 32 from resting eggs and 17 from clonally propagated lines, established from individuals sampled in the water column or hatched from resting eggs (fig. 1*A* and supplementary table S2, Supplementary Material online). Data gained from clonal lines with a species attribution were used as “parental species” data: five samples for *D. galeata*, four for *D. longispina*, and three for *D. cucullata*. The parental clones are part of two larger clone panels representing the parental species and their diversity in several European lakes. Their identity was established prior to this study either based on mitochondrial and microsatellite markers (M5, LC3_6, J2, Herrmann et al. 2017) or morphological examination, mitochondrial markers and factorial correspondence analyses based on microsatellite markers (Möst 2013; Alric et al. 2016), including hybrids and historical resting eggs, which separates parental species and hybrids (Dlouha et al. 2010; Rellstab et al. 2011; Yin et al. 2014; Alric et al. 2016). In addition, data were available for four resting eggs from Arendsee (AR), 12 resting eggs from Dobersdorfer See (DOB), five clonal lines and eight resting eggs from Eichbaumsee (EIC), and eight resting eggs from Selenter See (SE) (supplementary table S2, Supplementary Material online). Although the analysis of eggs from older sediment layers was attempted, biological material was either limited, of poor quality, or contaminated. Our isotope dating for DOB was unconclusive: either slides of the cored location or a high sedimentation rate meant the top 30 cm of the core did not show the usual isotope peaks, thus preventing precise dating. EIC samples were recent since they were collected from surface bank sand. For SE, the oldest eggs analyzed here originated from the 2–3 cm layer of the core, which corresponds to max. ∼17 years (Andersen T, personal communication). For AR the oldest eggs for which results were obtained were isolated from the 4–5 cm layer, corresponding to ∼2005 (Balínt M, personal communication).

An average of 89.9% (range: 31.7–98.6%) reads aligned to the reference genome with a mean coverage of 10.26*×* (range: 0.34–52.30*×*) (supplementary table S3, Supplementary Material online). The final SNP data set for subsequent analyses after quality-filtering included 3,240,339 SNPs across the 49 samples. To rule out possible reference bias, we compared mapping rates of reads with the reference allele and to the alternative allele at heterozygous sites. We found no preferential mapping of the reference allele, as all species categories and the hybrids had a median distribution close to 0.5 (supplementary fig. S2, Supplementary Material online).

### Principal Component Analysis

In a PCA including all genotypes and conducted with SNPRelate v1.20.1 (Zheng et al. 2012), the parental species genotypes grouped in three very distinct clusters. *Daphniacucullata* separated from *D. galeata* along PC1, which explained 7% of the variation. *Daphnialongispina* separated from *D. galeata* and *D. cucullata* along PC2 which explained 6% of the variation (fig. 1*B*). Although sampled in different lakes, all parental species genotypes were grouped in tight clusters along the two axes with little evidence for population substructure. Population AR clustered with the *D. cucullata* reference individuals while population samples from DOB, EIC, and SE were more spread out, mostly between the *D. galeata* and *D. longispina* clusters.

### Admixture Analyses Uncover Hybrids

The PCA results are confirmed by an admixture analysis conducted with ADMIXTURE (Alexander and Lange 2011) with *K* = 3, supported by the lowest cross-validation error of the tested K values. The known parental species genotypes were clearly separated into three clusters (fig. 1*C*). Although we detect no evidence of admixture in the AR and DOB samples and, based on our parental species, consider them to belong to the species *D. cucullata* and *D. galeata*, respectively, the two other populations seem to consist mostly of admixed individuals. The five EIC samples sequenced after clonal propagation were all found to be admixed (*D. galeata* and *D. longispina*), whereas the EIC resting eggs were either admixed (3) or belonged to one of the parental species (5). SE resting eggs present all combinations of admixture except *D. cucullata×D. longispina*: *D. galeata* (2), admixed between *D. galeata* and *D. cucullata* (3), and admixed between *D. galeata* and *D. longispina* (3).

### Ancestry Painting

**Fig. 2.**
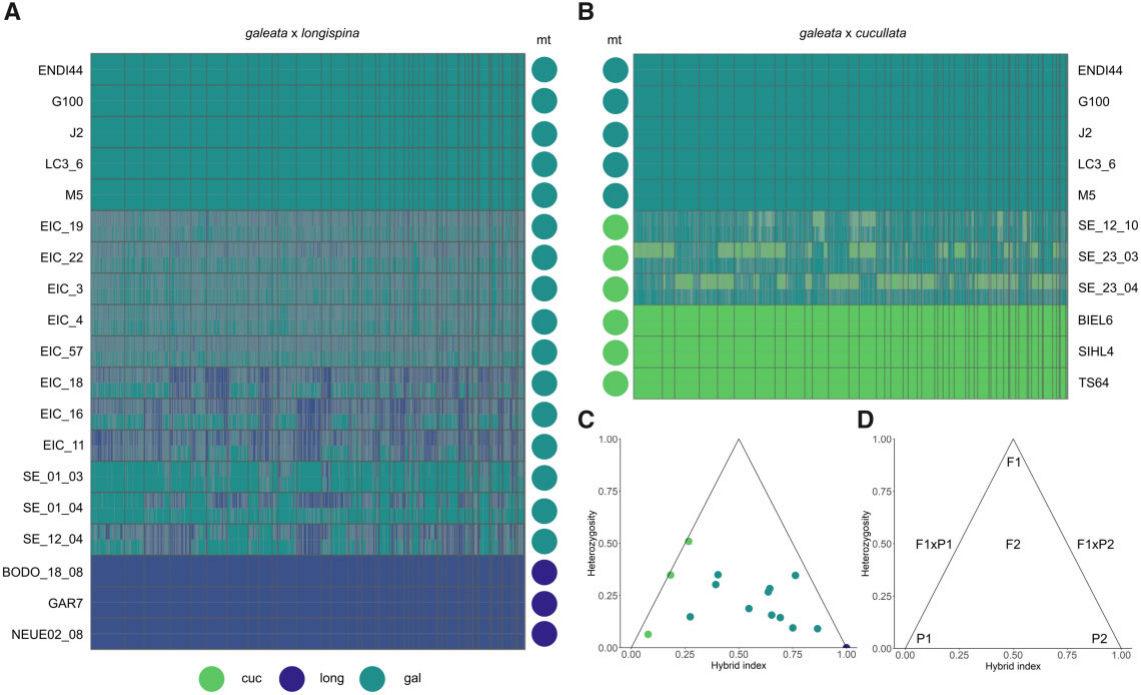
(*A*, *B*) Ancestry painting of the hybrid individuals identified through the admixture analysis. Each row represents an individual. Colored circles on the side indicate the mitochondrial identity of the individuals, based on the analysis of full mitochondrial genomes. Scaffolds are sorted by length and separated by thin grey lines. In panels (*A*) and (*B*), the five upper rows represent individuals assigned to the parental species *Daphnia galeata*. In (*A*), the last three rows correspond to individuals assigned to the parental species *D. longispina.* In (*B*), the last three rows correspond to individuals assigned to the parental species *D. cucullata.* Triangle plots summarizing (*C*) the hybrid index and mitochondrial species identity for all individuals identified as admixed, and (*D*) The hypothetical expected means of parental species (P1 and P2) and hybrid classes (F1xP1 and F1xP2: backcrosses with parental species P1 and P2, respectively).

Based on results obtained in the ADMIXTURE analysis, two pairs of species and their putative hybrids were analyzed with an “ancestry painting” approach, outlined in Barth et al. (2020) and Runemark, Eroukhmanoff, et al. (2018): *D. galeata* and *D. longispina* parental genotypes and putative hybrids between them from populations EIC and SE, and *D. galeata* and *D. cucullata* parental genotypes and putative hybrids between them from population SE. Briefly, after identifying fixed sites for each of the species in the analyzed pair, heterozygosity was calculated for these sites and a hybrid index derived from the obtained results (https://github.com/mmatschiner/tutorials/tree/master/analysis_of_introgression_with_snp_data, last accessed November 2021). Further, information on the maternal species is used to tentatively categorize the admixed individuals. For a first-generation hybrid (F1) the expectation would be 50% of the nuclear genome being derived from each parental species (hybrid index ≈ 0.5) and mostly heterozygous fixed sites (heterozygosity ≈ 1.0). Individuals originating from the backcrossing of F1 with one of the parental species are expected to have hybrid index values around 0.25 or 0.75 (fig. 2*D*). We consider individuals with intermediate hybrid indices (>0.25 and <0.75) and lower heterozygosity (<0.5) to be later-generation hybrids, meaning they have one or multiple hybrid ancestors we are not able to classify further (Slager et al. 2020) We consider individuals with a hybrid index of ≤0.25 or ≥0.75 to be backcrossed with the respective parental species in at least one generation and the majority of the genome derives from one species. This definition is broad and will be refined with the addition of a greater number of parental genotypes.

The comparison of genotypes from parental species *D. galeata* and *D. longispina* (five and three individuals, respectively) allowed identifying a total of 335,052 fixed sites between the two species. Due to the quality filters applied to parental and hybrid genotypes, we could analyze 131,914 of these fixed sites in the putative hybrids, where read coverage was sufficient. The diploid genotypes were then plotted for all hybrids as homozygous for either of the parental species or as heterozygous (fig. 2*A* for the 50 longest scaffolds). The *D. longispina* reference clone KL11 was excluded from further analysis due to issues with missing data. All 11 genotypes from SE and EIC identified as likely *D. galeata×D. longispina* hybrids in the ADMIXTURE analysis possessed a *D. galeata* mitochondrial genome. The proportion of the maternal *D. galeata* genome in these hybrids, however, varied greatly, between 27.4% and 86.6%, and they all showed low heterozygosity, between 9.1% and 34.6% (fig. 2*C* and table 2). These values are unlikely for F1 hybrids or backcrosses of F1 with one of the parental species (table 2).

**Table 2:** Data Derived from Ancestry Painting Analysis and Based on the Fixed Sites Inferred from Analyzing Parental Species Genotypes

Sample	Hybrid Index	Heterozygosity	Maternal Species	Hybrid	Interpretation
SE_12_10	0.079	0.064	cuc	gal×cuc	Backcross gal
SE_23_03	0.183	0.348	cuc	gal×cuc	Backcross gal
SE_23_04	0.267	0.509	cuc	gal×cuc	Unclear
EIC_19	0.653	0.156	gal	gal×long	Later-generation
EIC_22	0.644	0.283	gal	gal×long	Later-generation
EIC_3	0.751	0.095	gal	gal×long	Backcross gal
EIC_4	0.693	0.144	gal	gal×long	Later-generation
EIC_57	0.637	0.267	gal	gal×long	Later-generation
EIC_18	0.393	0.302	gal	gal×long	Later-generation
EIC_16	0.403	0.349	gal	gal×long	Later-generation
EIC_11	0.274	0.148	gal	gal×long	Later-generation
SE_01_03	0.866	0.091	gal	gal×long	Backcross gal
SE_01_04	0.763	0.346	gal	gal×long	Backcross gal
SE_12_04	0.547	0.187	gal	gal×long	Later-generation

Note.—Maternal species attribution is based on mitochondrial phylogeny, hybrid attribution is based on the ADMIXTURE plot.

Comparing genotypes from the parental species *D. galeata* and *D. cucullata* (five and three individuals, respectively) led to identifying 715,438 fixed sites between the two species (due to quality filtering, 275,216 of these sites were further analyzed). All three *D. galeata×D. cucullata* hybrids carried a *D. cucullata* mitochondrial genome, their hybrid index varied between 0.079 and 0.267 and their heterozygosity ranged from 6.4% to 50.9% (fig. 2*B* and *C*; table 2). The individual SE_23_04 is most likely the result of a backcrossing with *D. galeata*; however, it is difficult to determine what backcrossed with it: either an F1 hybrid or a later generation hybrid, that is, that resulted from several generations of admixture. Haplotype information would be needed to gain certainty. The other two hybrids’ lower hybrid index hints backcrossing with *D. galeata*, according to the criteria defined above.

### Population Genomics Parameters

**Fig. 3.**
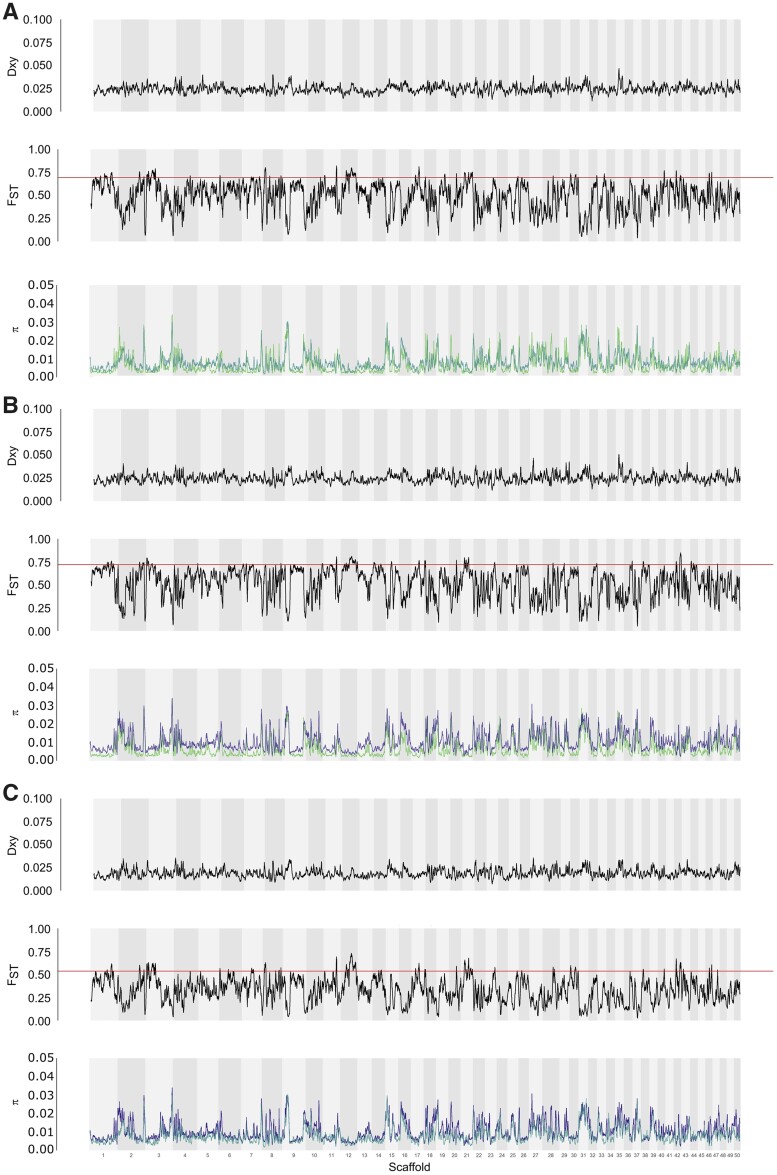
Window-based statistics for the pairs (*A*) *Daphnia galeata/D. cucullata*, (*B*) *D cucullata/D. longispina*, and (*C*) *D. galeata/D. longispina*, shown for the 50 largest scaffolds in 100-kb windows with 10-kb step size—calculations are for all nonadmixed individuals unambiguously assigned to parental species according to the ADMIXTURE analysis. In each panel from top to bottom: *d*_xy_ values, pairwise *F*_ST_ values with a red horizontal line indicating the 95th percentile, nucleotide diversity (*π*) for *D. galeata* (teal), *D. longispina* (dark blue), and *D. cucullata* (lime green).

To calculate genome-wide nucleotide diversity (*π*), between-taxon differentiation (*F*_ST_), and between-taxon divergence (*d*_xy_) within 100-kb sliding windows, we took advantage of the inference made with ADMIXTURE and pooled all genotypes which were unambiguously assigned to either of the parental species clusters. Consequently, a total of seven genotypes from four populations were classified as *D. cucullata*, eight from five populations as *D. longispina*, and 20 genotypes from eight populations as *D. galeata* (supplementary table S2, Supplementary Material online). All values (*d*_xy_, *π*, and *F*_ST_) were calculated with the script popgenWindows.py (github.com/simonhmartin/genomics_general, release 0.3, last accessed February 2021) for each species pair and are plotted for the 50 largest scaffolds in figure 3.

The window-based *F*_ST_ values for all three possible pairs among the three species averaged 0.274 for *D. galeata* versus *D. longispina*, 0.343 for *D. cucullata* versus *D. longispina* and 0.364 for *D. galeata* versus *D. cucullata*. The mean sequence divergence *d*_xy_ for the three pairs was 0.018 for *D. galeata* versus *D. longispina* and 0.022 for both *D. cucullata* versus *D. longispina* and *D. cucullata* versus *D. galeata*. Both parameters show similar patterns, with lower values on an average when comparing *D. galeata* to *D. longispina* than when comparing *cucullata* to either one of the other species. These patterns confirm the results obtained with other analyses, for example, the higher number of fixed sites between *D. galeata* and *D. cucullata* in the ancestry painting analysis.

The window-based estimates show high variability in levels of differentiation and divergence along the genome. Further, regions of high or low differentiation are mostly associated with depleted or high nucleotide diversity, respectively (see scaffolds 2 and 9, for example). However, the genome being represented by unordered scaffolds instead of chromosomes makes this difficult to interpret further.

Nucleotide diversity (*π*) to quantify the level of genetic variation within each taxon was on an average higher for *D. longispina* (1.18%) than for the other two species (0.95% and 0.85% for *D. galeata* and *D. cucullata*, respectively). This cannot be explained by the differences in group sample sizes, since *D. galeata* was the group with the largest sample size (and highest number of sampled populations). To ensure our window-based estimates were not biased because of the overrepresentation of some populations in a group (e.g., DOB in the *galeata* group), we also calculated these indices using only one individual from each population per species; if one population contained multiple individuals, we picked one individual at random to represent this population (see supplementary table S2, Supplementary Material online, for a listing of the used genotypes—results shown in supplementary fig. S4, Supplementary Material online).

Many more highly differentiated windows and genes were shared among two or all species pairs than would be expected by a random intersection (supplementary fig. S5, Supplementary Material online). For example, a total of 2,575 10-kb windows had an *F*_ST_ value within the 95th percentile in the pair *D. galeata*/*D. longispina* and 2,569 in the pair *D. galeata*/*D. cucullata*. The mean expected number of windows in common between these two pairwise comparisons was 113, but the number of windows in common observed in the data was 1,601. A similar pattern was observed in all other intersections. This result suggests that the location of differentiated genome parts is not due to random processes but has biological significance. A GO-enrichment analysis of these isolated genes to shed light on the function of these species-specific genes, however, was not possible, because of the low number of genes with GO annotation. For the pair *D. galeata/cucullata*, only 12% of the genes in the outlier windows were annotated with Gene Ontologies, for the pair *D. galeata/longispina* it was 11% and for the *D. cucullata/longispina* pair it was 10%.

### Phylogeny Based on Complete Mitochondrial Genomes

**Fig. 4.**
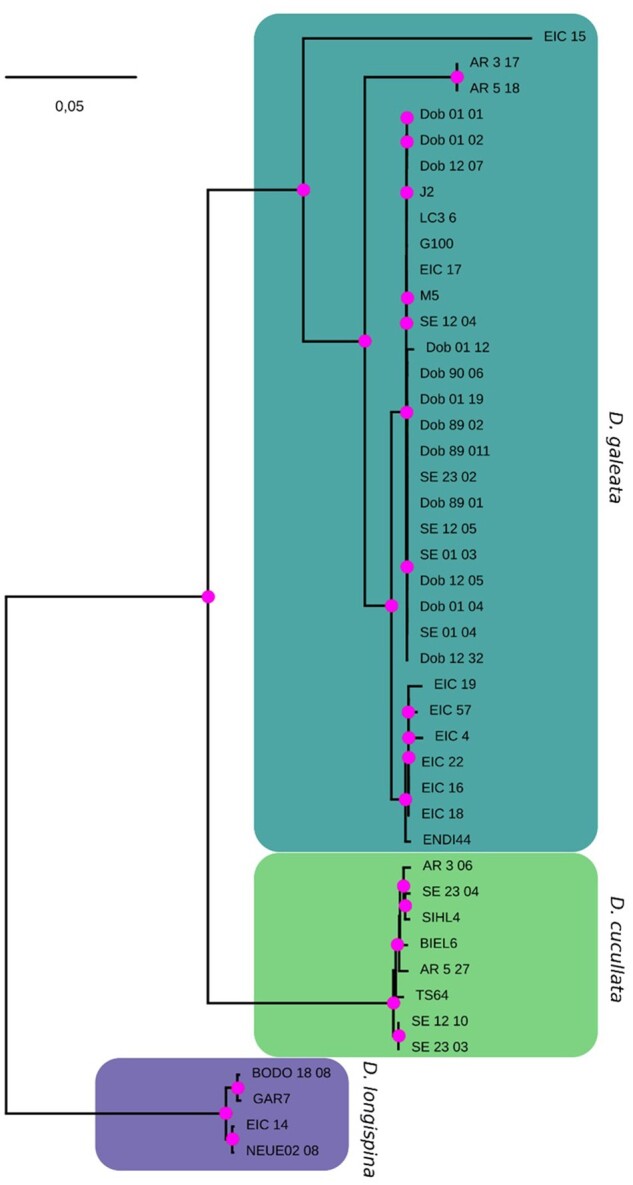
Maximum-likelihood tree reconstructed from mitochondrial PCGs and rRNA genes of parental species, clones sampled in the water column and resting eggs sequenced in this study. The tree reveals distinct and highly supported clusters corresponding to *Daphnia galeata*, *D. cucullata*, and *D. longispina* mitotypes (as defined by the respective parental species and a sister taxa relationship between *D. galeata* and *D. cucullata)*. Here, the best tree (log*L*=−47,950.82) rooted with outgroup *D. laevis* is depicted. Magenta dots indicate Shimodaira–Hasegawa approximate likelihood ratio test values ≥80% and ultrafast bootstrap support values ≥95% calculated from 10,000 bootstrap replicates (SH-aLRT/UFboot). The scale bar corresponds to 0.05 nucleotide substitutions per nucleotide site. KL11 was excluded due to missing data.

Phylogenetic reconstruction based on the mitochondrial protein-coding and ribosomal RNA (rRNA) genes were largely consistent with earlier mitochondrial phylogenies based on single or few mitochondrial genes (Adamowicz et al. 2009; Petrusek et al. 2012). We identified highly supported clades comprising the respective parental genotypes, hence representing *D. longispina*, *D. cucullata*, and *D. galeata* mitochondrial haplotypes (fig. 4). *Daphniacucullata* and *D. galeata* mitochondrial haplotypes clustered as sister groups. Although the mitochondrial haplotypes in the *D. longispina* and *D. cucullata* clusters do not show much divergence, the *D. galeata* haplotype cluster also contains deeper branching events (haplotype EIC_15 and AR3_17/AR5_18). Further, although all samples from AR were unequivocally categorized as *D. cucullata* in the ADMIXTURE analysis and clustered with *D. cucullata* parental genotypes in the PCA, two of them have mitochondrial haplotypes falling into the *D. galeata* cluster (AR3_17 and AR5_18). A similar mismatch was also observed for EIC_15, which clusters with *D. longispina* when considering nuclear SNP and with *D. galeata* when considering the mitochondrial genome. Within the species clusters, we observed a grouping by lake with many haplotypes being either identical or very similar when originating from the same location. The trees obtained with either only protein-coding genes (PCGs) (CODON model) or PCGs and rRNA genes but with a mixed model (DNA for rRNAs and CODON for DNA) were all consistent with the tree shown here and are therefore not included.

### Patterns of Introgression

We tested all four northern Germany populations (EIC, DOB, AR, and SE) for admixture between the three reference species with *f*_3_ statistics tests (table 3) and considered a *Z* score<−3 as significant (following Reich et al. 2009; Patterson et al. 2012). Negative and significant values (*f*_3_ = −0.19) using EIC as the test population and *D. galeata* and *D. longispina* as the source populations indicated mixed ancestry from these two or closely related populations. For population SE, the *f*_3_ test supports both admixed ancestry from *D. galeata* and *D. longispina* (*f*_3_ = −0.09) and *D. galeata* and *D. cucullata* (*f*_3_ = −0.15). All tests for population DOB and AR were positive providing no evidence of admixture events. The supported introgression events are consistent with the results in our previous analyses conducted with ADMIXTURE and the ancestry painting approach.

**Table 3:** Summary of the *f*_3_ Statistic for Admixture in the Form (C; A, B)

Source Population A	Source Population B	Target Population C	*f*_3_	SE	*Z*-Score	No. Sites
gal	long	AR	5.48226	0.154392	35.509	1,072,106
gal	cuc	AR	0.245124	0.006208	39.487	791,570
long	cuc	AR	0.225393	0.005475	41.165	875,293
gal	long	Dob	0.152725	0.003233	47.242	752,265
gal	cuc	Dob	0.150027	0.003028	49.546	815,035
long	cuc	Dob	1.763147	0.049657	35.506	1,059,418
gal	long	EIC	−0.193114	0.003224	**−59.898**	864,328
gal	cuc	EIC	0.086614	0.002623	33.025	1,119,715
long	cuc	EIC	0.014322	0.001774	8.071	1,145,530
gal	long	SE	−0.093233	0.003222	**−28.939**	1,029,744
gal	cuc	SE	−0.154696	0.003924	**−39.426**	955,516
long	cuc	SE	0.288212	0.011203	25.727	1,092,186

Note.—A significantly (*Z* score<−3, in bold) negative *f*_3_ value implies that the target population C is admixed. SE, standard error.

**Fig. 5.**
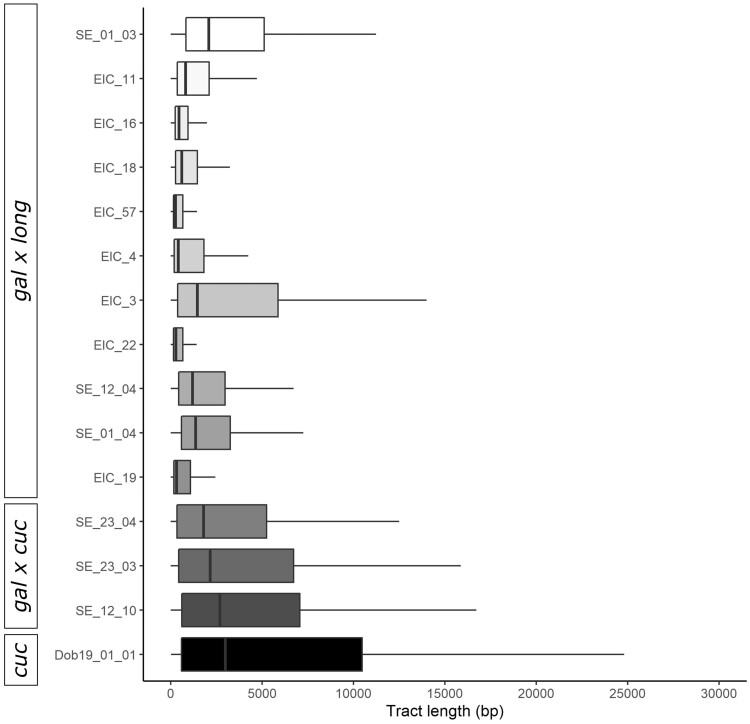
Distribution of the ancestry tract length where each ancestry tract represents the state of a SNP changing to the other species or the end of a scaffold in the local ancestry inference for each admixed individual and one nonadmixed *D. cucullata* individual. The nonadmixed individual displays the ancestry tract length distribution when all scaffolds derive from the same species. Hybrid type (according to ADMIXTURE analysis) is given on the left side.

We performed local ancestry inference with Loter (Dias-Alves et al. 2018) to trace genome-wide introgression among the hybrids and infer additional details about the parental species and backcross history from haplotype information. The results were summarized genome-wide for the ancestry proportion, heterozygosity of ancestry and the number of ancestry transitions where each ancestry tract is counted when the state of an SNP changes to the other species or at the end of a scaffold (fig. 5). The three *D. galeata×cucullata* hybrids were all found to have high *galeata* ancestry (73.9–94.2%) and the individuals SE_23_03 and SE_23_04 were confirmed as the offspring of a later-generation hybrid and a pure *galeata* parent (supplementary fig. S6*B*, Supplementary Material online).

Seven *D. galeata×longispina* hybrids had very high *galeata* ancestry (81.4–97.9%), and visual inspection of the ancestry tracts (supplementary fig. S6*A*, Supplementary Material online) revealed very short *longispina* tracts and scaffolds with multiple breakpoints indicating multiple generations of recombination. Four *D. galeata×longispina* hybrids had lower *galeata* ancestry (27.7–59.0%) and the presence of complete *longispina* scaffolds implying some backcrossing with *longispina*. The haplotype phasing confirmed that the parents of all *D. galeata×longispina* hybrids were also of hybrid origin. The average and maximum ancestry tract length for all *D. galeata×longispina* hybrids is shorter than those for *D. galeata×cucullata* hybrids.

## Discussion

### A Reference Genome for Studying a Species Complex

*Daphnia* are a key species in freshwater habitats. Previous studies have established reference genomes for the model species *D. pulex* (Colbourne et al. 2011; Ye et al. 2017) and *D. magna* (Lee et al. 2019). No high-quality reference genome for species belonging to the DLSC was available so far. To date, it is unclear whether the ecological differentiation and/or intrinsic incompatibilities drive and maintain divergence between DLSC species. Besides its utility for studies of hybridization events in the DLSC, the new assembly we present here will thus allow us to better understand the evolution of a key species in European freshwaters.

Even though the onset of the DLSC radiation was dated to 5–7 Ma based on nuclear and mitochondrial markers (Schwenk 1993; Taylor et al. 1996; Adamowicz et al. 2009), several factors confirm the suitability of this reference for all tested species. Mapping success and coverage of whole-genome data from *D. cucullata* and *D. longispina* to the reference genome were high, and we found no evidence of reference bias. This assembly clearly benefited from advances both in the sequencing technologies and assembly and postprocessing algorithms since the first *Daphnia* genome (Colbourne et al. 2011). The metrics used for assessing its quality reveal that in particular, the combination of long- and short-read technologies led to highly contiguous and accurate scaffolds. Although we likely did not recover the genome in its full length (133 Mb out of an estimated 156 Mb), and the N50 value is lower than those obtained for *D. pulex* (Ye et al. 2017) and *D. magna* (Lee et al. 2019), iterative scaffolding allowed for a very efficient gap-closing, and an exceptionally low number of mismatches, compared with the other *Daphnia* assemblies.

### Pervasive Introgression in the *DLSC*

We utilize a method that allows us to interrogate biological archives and analyze whole *Daphnia* genomes directly from the resting egg bank (Lack et al. 2018) without hatching and culturing several clonal lineages. This provides a wide sweep of populations, past and present, with each egg being the product of local sexual recombination.

Although no evidence of introgression was found in the DOB population, the three other locations host a variety of admixed genotypes. SE & EIC can even be considered hybridization hotspots with more than 60% of individuals having hybrid ancestry, as revealed in the ADMIXTURE analysis. However, Kong and Kubatko (2021) very recently showed that ADMIXTURE is sensitive to unequal contributions by the parental species, and we thus sought to support these inferences by *f_3_* calculations and using an ancestry painting approach.

In DOB, ADMIXTURE delivered unequivocal results. Further, the *f_3_* index indicated that no introgression was detectable in this population. However, the PCA plot shows that some of the DOB genotypes are near hybrid individuals. ANGSD results are similar but these genotypes nearer the parental species (supplementary fig. S3*A*, Supplementary Material online). To address these slightly contradictory results, we therefore conducted an ancestry painting on two DOB genotypes, (12_07 and 89_02, supplementary table S7, Supplementary Material online). Both genotypes had a very low heterozygosity, thus confirming the ADMIXTURE and *f_3_* outcomes. A possible explanation would be that these two genotypes carry variation that is not reflected in our limited sampling of the parental species. When comparing with microsatellite-based analysis including many more populations and data points (Thielsch et al. 2009), the *D. galeata* cluster has often been larger and more diverse than the others. The seemingly two “stray” DOB genotypes are therefore likely well within the species variation boundaries. All mitochondrial haplotypes were clustered together in the phylogenetic reconstruction as well.

In AR, despite the high resting egg density found in the sediment, only very few could be successfully genotyped. Although all inferences based on nuclear markers (PCA, ADMIXTURE, *f_3_*) indicated an absence of hybridization or introgression in this population, the mitochondrial phylogenetic reconstruction showed diverging results. From a nuclear point of view, all genotypes could be categorized as *D. cucullata*, but two out of four AR individuals presented the mitochondrial genome of another species, that is, *D. galeata*. However, the phylogenetic reconstruction shows that the two AR mitochondrial haplotypes form a cluster separate from the main *D. galeata* cluster, which hints at different evolutionary history for these mitochondrial genomes. Such distinct lineages within a species and mito-nuclear discordances were also found by Thielsch et al. (2017) in the DLSC and mitochondrial capture has been detected in other *Daphnia* species (Marková et al. 2013). It is an interesting phenomenon in the DLSC that merits to be further investigated in the future with broader sampling.

In EIC, both pelagic samples and resting eggs were analyzed. Genotypes sampled alive from the water column were all inferred to be admixed to various degrees, three resting egg samples were also admixed, and the remaining five were assigned to either one of the two parental species. We conducted the ancestry painting approach on all EIC individuals; the fixed sites heterozygosity of the individuals categorized as nonadmixed in ADMIXTURE was indeed near zero (supplementary table S7, Supplementary Material online). Such high abundances of *D. galeata×longispina* hybrid resting eggs in periods of rapidly changing environmental conditions (i.e., eutrophication) have also been recorded in Lake Constance (Brede et al. 2009). The high frequency of *D. galeata×longispina* hybrids observed here might be due to similar ecological history: the lake Eichbaumsee was created through sand excavation for construction work around ∼40 years ago and is characterized by extreme eutrophication and even hypertrophy that could not be remediated. The presence of later-generation hybrids and backcrosses with *D. galeata* and *D. longispina* and short ancestry tract length suggest that both species have been present and hybridizing for most of the lake’s short history, or even that it was colonized by individuals of hybrid origin. However, we only obtained contemporary samples for EIC and analysis of resting eggs from sediment cores are needed to distinguish between the two hypotheses.

In SE, diversity is high, both in terms of species combinations in admixed individuals and in terms of degrees of introgression. Although we analyzed eggs from sediments cores, they originated from the first centimeters and there is, therefore, no clear temporal pattern that separates the different hybrid combinations found here. Strikingly, although SE and DOB are geographically very close to each other (∼10 km), and dispersal of resting eggs through, for example, waterfowl or storms would be possible (Figuerola et al. 2005; Pietrzak and Slusarczyk 2006; Frisch et al. 2007), the *Daphnia* communities are quite different. This might be due to their different eutrophication levels, reflect the fact that initial colonization was followed by the establishment of different species, or a combination of both. The observed diversity at such a small spatial scale underlines the mosaic nature of freshwater habitats and the usefulness of approaches including many populations to fully understand genetic diversity arising from colonization and hybridization events in the DLSC.

Previous studies using mitochondrial and few nuclear markers (Thielsch et al. 2012; Alric et al. 2016) were able to categorize hybrids into F1, F2, and backcrosses. However, due to the low resolution of the used markers, further categorizing and above all identification of genome-wide breaking points was not possible at the time. The *D. galeata* reference genome and resequencing data offer now a much higher resolution to assess later generation hybrids and patterns across the genome. In general, hybrids identified in this study seem to have a complex history of multiple generations of hybridization and backcrossing with both parental species that we are not able to detangle using only ancestry paintings. The local ancestry inference revealed that the average ancestry tract length for *D. galeata×longispina* hybrids from EIC and SE is shorter than those for *D. galeata×cucullata* hybrids. There are several explanations for the observed pattern. One is that more generations of recombination led to shorter introgressed tracts, and the *D. galeata×longispina* hybrids are therefore the result of a greater number of sexual generations than the *D. galeata×cucullata* hybrids. The genomic mosaic of ancestry segments for all hybrid individuals is also characterized by multiple breakpoints within the same scaffolds, which is only possible after multiple generations of recombination. However, data on genome-wide recombination rates and selection are needed to reach solid conclusions about the correlation between tract length and age of the hybridization event in the individual’s ancestors. Alternatively, reproductive isolation might be lower between *D. galeata* and *D. longispina* than between *D. galeata* and *D. cucullata*, thus leading to faster introgression in the former case.

As evidenced by the comparison of genomic windows of higher divergence between species pairs, the introgression pattern is not random: a given region exhibiting high *F*_ST_ values between the *D. galeata* and *D. longispina* genotypes is also likely to show similarly high *F*_ST_ values in the *D. galeata*/*D. cucullata* pair. Further, some parts of the genome seem to be effectively shielded from introgression. About a quarter of all genes (4,136) are in regions that are highly differentiated between at least two species and about 5% (859) in parts of the genome that are isolated among all three species of the complex. This is much more than expected by chance (supplementary fig. S3, Supplementary Material online) and is thus likely due to selection against introgression. It seems plausible to search among these for genes that conserve the specific identity of the involved taxa, despite incomplete reproductive isolation. Genes responsible for the observed ecological divergence among the taxa (Schwenk et al. 2000) or genetic incompatibilities are most likely candidates to be found in the observed divergent regions. Given the ancient divergence, the speciation process in the DLSC might have attained a selection–migration–drift equilibrium, for which there is growing empirical evidence in other species like stick insects (Riesch et al. 2017), flycatchers (Burri et al. 2015), and nonbiting midges (Schreiber and Pfenninger 2021). However, the current snapshot could equally likely be a consequence of one or several pulses of hybridization. To assess the stability of the equilibrium, data showing that the introgression/selection process is ongoing and constant across an extended period of time would be required and *Daphnia* offers the unique opportunity to go back in time to test these alternative hypotheses.

### New Evidence for Cytonuclear Discordance

The genome-wide perspective also elucidated discordance between nuclear and mitochondrial patterns. The phylogeny based on mitochondrial genomes conforms to previously inferred relationships in the DLSC and suggests *D. galeata* and *D. cucullata* are sister species, with *D. longispina* as an outgroup (Adamowicz et al. 2009; Petrusek et al. 2012). However, several of our analyses based on nuclear SNPs challenge this view and suggest different evolutionary histories for mitochondrial and nuclear genomes. The ancestry painting approach relies on the identification of fixed sites for species in a pairwise comparison. More sites were found to be fixed between *D. galeata* and *D. cucullata* (715,438) than between *D. galeata* and *D. longispina* (335,052), which implies a greater divergence between members of the former pair. Further, *F*_ST_ values were on an average higher between *D. galeata* and *D. longispina* (0.274) than between *D. galeata* and *D. cucullata* (0.364). Reports of cytonuclear discordance are common both in plants (Huang et al. 2014; Stephens et al. 2015; Folk et al. 2017; Lee-Yaw et al. 2019) and animals (Llopart et al. 2014; Melo-Ferreira et al. 2014; Sarver et al. 2021). Several processes can lead to this discordance among closely related species: incomplete lineage sorting causing phylogenetic reconstructions based on mitochondrial markers to differ from the true phylogeny of the taxa, or selection causing the fixation of different mitochondrial genomes in different places from standing variation within species (Barrett and Schluter 2008). Alternatively, cytonuclear discordance may reflect hybridization between species and cytoplasmic introgression, accompanied or not by selection (reviewed in Sloan et al. [2017]). The latter explanation would be quite conceivable in the DLSC.

## Conclusion

We here provide the first high-quality resources to study genome-wide patterns of divergence in the DLSC, an ecologically important taxon in European freshwater habitats. By quantifying intra- and interspecific diversity, we provide a first glimpse into introgressive hybridization and lay the ground for further studies aiming at understanding how species boundaries are maintained in the face of gene flow.

Unlike for *D. pulex* and *D. magna*, no linkage groups are known for any species of the DLSC. Hi-C sequencing data will be added in the future to order scaffolds into larger, potentially chromosome-scale scaffolds. Such an approach holds promise in a species complex were laboratory crossings for F2 panels and traditional mapping are nearly impossible. This will allow discovering structural variants, identifying recombination breakpoints along each chromosome and thus provide a deeper understanding of the introgression patterns observed here. The functional role of genes in the regions of high divergence uncovered through this first analysis is yet unclear and will be addressed in future studies.

Finally, wider sampling, with the inclusion of more populations as well as more members of the species complex, and the reconstruction of a nuclear based phylogeny are necessary to reach conclusions about the species relationships and eventually identify the causes of the pattern uncovered here.

## Materials and Methods

### Sampling

The clonal line used for genome sequencing and assembly, M5, was hatched from a resting egg isolated from the upper layers (first 5 cm, corresponding to the years 2000–2010) of a sediment core taken in Lake Müggelsee in 2010. Further, single genotypes representing the parental species from various locations were used in this study, henceforth “parental species genotypes.” Most of them were established from individuals sampled from the water column and are still maintained through asexual reproduction as monoclonal cultures in the laboratory. Thus, all individuals of a clonal line are the same genotype and can be pooled to achieve sufficient amounts of genomic DNA. The species identity for these genotypes was established through a combination of methods: morphology, mitochondrial sequences, and nuclear markers.

Sediment cores were collected from Dobersdorfer See (DOB), Selenter See (SE), and Arendsee (AR), Germany using a gravity corer (Uwitec, Mondsee, AT) (supplementary table S4, Supplementary Material online). Samples were taken from the deepest part of the lakes to minimize past disturbance of the sediment. Cores were cut horizontally into 1 cm layers and the layers were stored at 4 °C in the dark to prevent hatching. Sediment rate of the three lakes was determined using radioisotope dating (^137^Cs and ^210^Pb).

In addition, lake sediment from the shoreline of Eichbaumsee (EIC) (supplementary table S4, Supplementary Material online), Germany was collected by hand and stored at 4 °C. The exact age of the sediment is unknown but the upper layers most likely contain recent eggs from the last few years. Zooplankton samples were taken from Eichbaumsee using a plankton net (mesh size 150 µm) from which six *Daphnia* clonal lines were established in a laboratory setting with artificial medium (Aachener Daphnien Medium, ADaM Klüttgen et al. 1994).

All sampling locations are plotted in figure 1*A* and information on all samples is provided in supplementary table S2, Supplementary Material online.

### Genome Sequencing

#### DNA Extraction for Genome Sequencing with Illumina and PacBio

DNA was extracted from around 60 clonal M5 individuals collected from batch cultures maintained in ADaM, and fed with the algae *Acutodesmus obliquus*, cultivated in medium modified after (Zehnder and Gorham 1960). Extraction was conducted following a phenol chloroform-based protocol with an RNase step and subsequently sequenced on an Illumina HiSeq4000 at BGI China. Additionally, tissue samples with around 3,000 individuals were sent to BGI for DNA extraction and PacBio sequencing.

### Resequencing (Population Genomics Approach)

#### DNA Extraction from Batch Cultures for Resequencing

For clonal lines used as reference for the parental species, individuals were raised in batch cultures and treated with antibiotics prior to collection and storage at −20 or −80 °C. DNA was extracted with either a phenol chloroform method, a (modified) CTAB protocol or a rapid desalting method (MasterPure Complete DNA and RNA Purification Kit; Lucigen Corporation).

Total genomic DNA was isolated from 20 pooled adult *Daphnia* for each of the five EIC clonal lines using a CTAB extraction method (Doyle and Doyle 1987).

#### Whole-Genome Amplification on Resting Eggs for Resequencing

To isolate *Daphnia* resting eggs from the sediment each sediment layer was sieved using a sieve with 125 µm mesh size and small amounts of the remaining sediment were resuspended in distilled water. Ephippia were eye spotted under a stereomicroscope, counted, and transferred to 1.5 ml tubes. The water was removed and ephippia stored at −20 °C in the dark until further analysis.

The ephippia were then opened under a binocular with insect needles and tweezers previously treated under a clean bench (UV sterilization) and with DNase away (Thermo Fisher). Eggs that were already damaged, had an uneven shape or were orange, which is evidence for degradation, were discarded. The resting egg separated from the ephippial casing washed in 15 µl sterile 1*×* PBS and then transferred in 1 µl 1*×* PBS to a new tube with 2 µl fresh 1*×* PBS. The isolated eggs were stored at −80 °C at least overnight.

For WGA of single eggs, the REPLI-g Mini Kit (Qiagen) was used. This kit is enabling unbiased amplification of genomic loci via Multiple Displacement Amplification. The isolated resting eggs were thawed on ice and the whole genome was amplified following the manufacturer’s protocol for amplification of genomic DNA from blood or cells. Briefly, denaturation buffer was added to the prepared resting eggs in 3 µl 1*×* PBS and amplified by REPLI-g Mini DNA Polymerase under isothermal conditions for 16 hours.

The amplified product was quantified on a Nanodrop spectrophotometer (Thermo Fisher) and with a Qubit Fluorometer (Thermo Fisher). Successful amplifications were purified with 0.4*×* Agencourt AMPure XP magnetic beads (Beckman Coulter) to remove small fragments and eluted in 60 µl 1*×* TE buffer.

Fragments of the mitochondrial gene 16S rRNA gene were amplified to check successful amplification of *Daphnia* DNA using the universal cladoceran primers S1 and S2 (Schwenk et al. 1998) and a low presence of bacterial DNA using universal primers for the bacterial 16S rDNA gene (Nadkarni et al. 2002). Only samples with a successful amplification of the *Daphnia* 16S fragment and low amplification of the bacterial 16S fragment indicating low bacterial contamination were used for sequencing steps.

#### Library Preparation and Sequencing of Resequencing Samples

After quantification and quality control of the DNA using Nanodrop and Qubit instruments, libraries were prepared either directly in-house with the NEBNext Ultra II DNA Library Prep Kit for Illumina (New England Biolabs), or at the sequencing company Novogene (Cambridge, UK). Resequencing (paired-end 150-bp reads) was then performed either at Novogene (UK) Company Limited or the Functional Genomics Center (ETH Zurich and University of Zurich) on Illumina NovaSeq 6000 and HiSeq4000 instruments.

Details on the procedure used for each sample are provided in supplementary table S2, Supplementary Material online.

### Genome Assembly and Annotation

We provide here a summarized version of the procedure used to assemble and annotate the genome. Details can be found in supplementary methods, Supplementary Material online.

#### Raw Data QC

Illumina reads were trimmed and the adapter removed using a combination of Trimmomatic 0.38 (Bolger et al. 2014), FastQC 0.11.7 (Andrews 2010) and MultiQC 1.6 (Ewels et al. 2016) within autotrim 0.6.1 (Waldvogel et al. 2018). To filter out reads possibly originating from contamination from known sources (see below), a FastQ Screen like approach was chosen. In brief, the reads are separated by results of mapping behavior to different genomes. Positive controls consisted of genome data for other *Daphnia* species (dmagna-v2.4 and Daphnia_pulex_PA42_v3.0, see supplementary methods, Supplementary Material online, for accession numbers), and negative control, that is, sequences deemed undesirable for genome assembly consisted of genome data from human, bacteria, viruses, and the algae used to feed the batch cultures. The resulting database comprised 108,163 sequences (total sequence space 42.2 Gb). Both Illumina reads and PacBio subreads were mapped against the database with NextGenMap (Sedlazeck et al. 2013) and minimap2 (Li 2018), respectively.

Reads did only pass the filtering if they either did not map to the database at all or had at least one hit against one of the two *Daphnia* genomes. Supplementary table S2, Supplementary Material online gives an overview of the effect of different filtering steps.

#### Assembly and Contamination Screening

All paired and unpaired contamination filtered Illumina reads as well as the contamination filtered PacBio reads were used as input for RA 0.2.1 (https://github.com/rvaser/ra, last accessed November 2021). Blobtools 1.0 (Laetsch and Blaxter 2017) was used to screen the resulting assembly for possible unidentified contamination in the hybrid assembly. Briefly, bwa mem 0.7.17 (Li 2013) was used to map Illumina reads back to the assembly and taxonomic assignment was done by sequence similarity search with BlastN 2.9.0+ (Camacho et al. 2009). Contamination with different bacteria was clearly identifiable, and contigs with coverage below 10*×* and/or GC content above 50% were removed. Additionally, PacBio reads mapping to these contigs were removed to minimize false scaffolding in further steps. The contig corresponding to the mitochondrial genome was identified after a BLAST search against available mitochondrial genomes for this species and removed from the assembly.

#### Scaffolding and Gap Closing

The blobtools filtered PacBio reads were used for scaffolding and gapclosing, which was conducted in three iterations. Each iteration consisted of a scaffolding step with SSPACE LongRead 1-1 (Boetzer and Pirovano 2014), a gap closing step with LR Gapcloser (https://github.com/CAFS-bioinformatics/LR_Gapcloser; commit 156381a, last accessed November 2021), and a step to polish former gap parts with short reads using bwa mem 0.7.17-r1188 and Pilon 1.23 (Walker et al. 2014) in a pipeline developed to this effect, wtdbg2-racon-pilon.pl 0.4 (https://github.com/schellt/wtdbg2-racon-pilon, last accessed November 2021).

#### Assembly Quality Assessment

Contiguity was analyzed with Quast 5.0.2 (Gurevich et al. 2013) at different stages of the assembly process. Further, mapping rate, coverage, and insert size distribution were assessed by mapping Illumina and PacBio reads with bwa mem and Minimap 2.17 respectively. To show absence of contamination in the assembly blobtools was ran as above. The genome size was estimated by dividing the mapped nucleotides by the mode of the coverage distribution of the Illumina reads by backmap 0.3 (https://github.com/schellt/backmap, last accessed January 2021), resulting in 156.86 Mb (with the obtained assembly length amounting to 85% of this estimated length). Additionally, the genome size was estimated using a k-mer based approach by creating a histogram from raw Illumina reads with Jellyfish 1.1.12 (Marçais and Kingsford 2011) and running the GenomeScope web application (http://qb.cshl.edu/genomescope/, last accessed November 2021) resulting in a genome size estimate of 150.6 Mb.

Completeness in terms of single copy core orthologs of the final scaffolds was assessed with BUSCO 3.0.2 (Simão et al. 2015), using the Arthropoda set (odb9).

#### Genome Annotation

RepeatModeler 2.0 (Smit and Hubley 2015) was run to identify *D. galeata* specific repeats. The 1,115 obtained repeat families were combined with 237 *D. pulex* and one *D. pulicaria* repeat sequences from RepBase release 20181026 to create the final repeat library. The genome assembly was then soft masked with RepeatMasker 4.1.0 (Smit et al. 2013–2015), resulting in 21.9% of the assembly being masked.

Gene prediction models were produced with Augustus 3.3.2 (Stanke et al. 2008), GeneMark ET 4.48_3.60_lic (Lomsadze et al. 2005), and SNAP 2006-07-28 (Korf 2004). The Augustus model was based on the soft masked assembly and the *D. galeata* transcriptome (HAFN01.1, Huylmans et al. 2016). The GeneMark model was obtained by first mapping trimmed RNAseq reads to the assembly with HISAT 2.1.0 (Kim et al. 2019) and then processing the resulting bam file with bam2hints and filterIntronsFindStrand.pl from Augustus to create a gff file with possible introns, which was finally fed into GeneMark.

The structural annotation was conducted in MAKER 2.31.10 (Holt and Yandell 2011). Briefly, the unmasked genome assembly, the species own transcriptome assembly as ESTs, the complete Swiss-Prot 2019_10 (UniProt Consortium 2019) and the protein sequences resulting from *D. magna* (Lee et al. 2019), as well as *D. pulex* (Ye et al. 2017) genome annotations as protein evidence, were used as input for MAKER. In total, three iterations of MAKER with retraining of the Augustus and SNAP model in between the iterations were conducted.

The quality of the structural annotation was assessed by comparing values as number of genes, gene space, etc. to existing annotations for other *Daphnia* genomes. Furthermore, core orthologs from BUSCO’s Arthropoda (odb9) set and conserved domain arrangements from the Arthropoda reference set of DOGMA 3.4 (Dohmen et al. 2016) were searched in the annotated protein set.

The functional annotation was conducted using InterProScan 5.39-77.0 (Jones et al. 2014) as well as a BLAST against the Swiss-Prot 2019_10.

### Population Samples

#### Raw Data QC and Contamination Check

The quality of raw reads was checked using FastQC v0.11.5. Adapter trimming and quality filtering were performed using Trimmomatic v0.36 with the following parameters: ILLUMINACLIP: TruSeq3-PE.fa: 2:30:10 TRAILING: 20 SLIDINGWINDOW: 4:15 MINLEN: 70. For samples sequenced on a NovaSeq6000 instrument and presenting a typical polyG tail, the program fastp (Chen et al. 2018) was used for trimming as well. To assess contamination in the WGA samples FastQ Screen v0.14.0 with the bwa mapping option was used (Wingett and Andrews 2018). A custom database was built to map trimmed reads against possible contaminants that included general common contaminants such as *Homo sapiens*, the UniVec reference database, a bacterial and a viral reference set as well as the *D. galeata* genome and *Acutodesmus obliquus* draft genome (see supplementary methods, Supplementary Material online, for accession numbers). Samples with <25% reads mapped to the *D. galeata* genome (and 25% contamination) were excluded from further analysis because the whole amplification of the resting egg most likely failed.

#### Mapping to Reference Genome and Variant Calling

The variant calling was performed within the Genome Analysis Toolkit (GATK v4.1.4.0; McKenna et al. 2010) program according to GATK4 best practices (Van der Auwera et al. 2013). The trimmed reads were mapped to the *D. galeata* genome using the BWA-MEM algorithm in BWA v0.7.17 with the -M parameter and adding read group identifiers for Picard compatibility (Li and Durbin 2009). PCR duplicates were marked and filtered out in the BAM file using Picard v2.21.1 (http://broadinstitute.github.io/picard/, last accessed November 2021).

To call variants for each sample GATK HaplotypeCaller in GATK was used with the –emitRefConfidence GVCF option resulting in a genomic variant call format (gVCF) file with information on each position for each individual (Poplin et al. 2018). All gVCF files were consolidated using CombineGVCFs and joint genotyping was performed with GenotypeGVCFs. The VCF file was filtered to include only SNPs and hard filtering was performed to remove variants with a QualByDepth <10, StrandOddsRatio >3, FisherStrand >60, mapping quality <40, MappingQualityRankSumTest <−8, and ReadPosRankSumTest <−5.

Subsequently, we removed sites with either very high coverage (>450) or for which genotypes were missing for more than 20% of the individuals using VCFtools v0.1.5 (Danecek et al. 2011). The final SNP data set for downstream analyses included 3,240,339 SNPs across the 49 samples.

In addition, GenotypeGVCFs was run with the –include-non-variant-sites option to output all variant as well as invariant genotyped sites. The final invariant data set included 127,530,229 sites after removing indels and multiallelic sites with BCFtools v1.9 (Li 2011) and is used for population genomic analysis to be able to calculate the total number of genotyped sites (variant and invariant) within a genomic window.

As we mapped all different species to the reference *D. galeata* genome, we assessed possible reference bias by checking the distribution of reference and alternative alleles observed at heterozygous genotypes based on Pinsky (2021). We pooled all genotypes which were unambiguously assigned to either of the parental species clusters *D. galeata*, *D. cucullata*, and *D. longispina* as was done for the population genomic parameters (supplementary table S2, Supplementary Material online) or classified as hybrids using ADMIXTURE inference. Without reference bias, we would expect that in heterozygous genotypes the reference and the alternative allele are on an average represented by 50% of the reads. An indication of reference bias would be that the *D. galeata* reference allele would be more frequent.

### Phylogenetic and Population Genetics Inferences

#### Mitochondrial Genome Assemblies and Phylogenetic Analyses

All reads were used to produce mitochondrial genome assemblies using the “de novo assembly” and “find mitochondrial scaffold” modules provided in MitoZ v2.4 with default settings (Meng et al. 2019). For some samples, this was not sufficient and we used two approaches to recover a complete mitogenome: either the mitochondrial baiting and iterative mapping implemented in MITObim v1.9.1 (Hahn et al. 2013) with the *D. galeata* mitochondrial reference genome or the modified baiting and iterative mapping in GetOrganelle v1.7.1 (Jin et al. 2020) with the animal database and k-mer values set to 21, 45, 65, 85, and 105. The procedure used for each data set is given in supplementary table S5, Supplementary Material online.

We annotated the mitochondrial genome assemblies with the mitochondrial annotation web server MITOS2 (Bernt et al. 2013) using the mitochondrial codon code 05 for invertebrates. Automated genome annotation identified 13 PCGs, two rRNA genes, and 22 transfer RNA genes (tRNAs). Initially, the mitochondrial genes (PCGs and rRNAs, supplementary table S6, Supplementary Material online) were individually aligned with MUSCLE v3.8.1551 (Edgar 2004) and visually checked for their quality. The mitochondrial genome assemblies with discrepancies, that is, a lot of missing data and/or split features were excluded from further analysis. The final data set (supplementary table S6, Supplementary Material online) included 44 mitochondrial genomes from this study and the previously published mitochondrial genome of *Daphnia laevis* (Martins Ribeiro et al. 2019, accession number: NC_045243.1). The mitochondrial genes of the final data set were individually realigned with MUSCLE v3.8.1551 (Edgar 2004) and MACSE v2.05 (Ranwez et al. 2018) and concatenated into a mitochondrial DNA matrix (supplementary table S6, Supplementary Material online) using SequenceMatrix v1.8.1 (Vaidya et al. 2011). During this step, we used MACSE v2.05 to realign PCG genes keeping the information about codon position (gene partitioning) and to remove STOP codons. The final data set consisted of the concatenation matrix of the 13 PCGs and the two structural rRNA genes. With this alignment, phylogenetic trees were reconstructed using IQ-TREE v1.6.12 (Nguyen et al. 2015). We initially partitioned the alignment into a full partition model, that is, each gene and all three codon positions for PCGs, and then ran IQ-TREE with partition analyses (-spp, Chernomor et al. 2016), ModelFinder (- m MFP+MERGE, Kalyaanamoorthy et al. 2017) and 10,000 ultrafast bootstrap (-bb 10,000, Hoang et al. 2018) and SH-like approximate likelihood ratio test (-alrt 10,000, Guindon et al. 2010) replicates. The resulting trees were visualized in R (R Core Team 2017) using the multifunctional phylogenetics package phytools (Revell 2012).

#### Ancestry and Population Structure

A principal component analysis was conducted in R v3.6.2 (R Core Team 2017) with the package SNPRelate v1.20.1 (Zheng et al. 2012). Linkage disequilibrium (LD) was calculated within a 500-kb sliding window and LD-pruned for *r*^2^ values >0.5 before conducting the PCA for all sites using the snpgdsPCA function with default settings. The relative large LD value was chosen because clonal reproduction and the overlap of generations due to diapause leads to increased LD in *Daphnia* (Brede et al. 2009).

Genetic admixture was estimated using ADMIXTURE v1.3.0 (Alexander and Lange 2011). The SNP set VCF file was converted to BED format using plink v1.90b6.13 (Chang et al. 2015). The log-likelihood values were estimated for one to five genetic clusters (K) of ancestral populations and admixture analysis were run for the most appropriate K value with 10-fold cross-validation. We also conducted the PCA and Admixture analysis using PCAngsd implemented in ANGSD and NgsAdmix, respectively (Korneliussen et al. 2014) to take genotype likelihoods into account (details in supplementary methods, Supplementary Material online). The results did not differ substantially and are shown in supplementary figure S3, Supplementary Material online.

However, using such a population genetic clustering approach to estimate ancestry coefficients is not directly equivalent to the proportion of hybrid ancestry in each individual and should be interpreted with caution (Lawson et al. 2018; Kong and Kubatko 2021). The results of the ADMIXTURE analysis suggested that the data set included hybrids between *D. longispina* and *D. galeata* as well as *D. cucullata* and *D. galeata*. We then followed the “ancestry painting” procedure outlined in Barth et al. (2020) and Runemark, Trier, et al. (2018), and classified sites according to their *F*_ST_ values when comparing parental species sets. Unlike the PCA and the admixture analysis, this approach requires the user to define parental genotypes; the individuals belonging to these sets are indicated with stars in figure 1*C*. Fixed sites are those where a specific allele is fixed in all individuals belonging to one parental species and another allele fixed in the other parental species. To show the ancestry of the hybrid individuals each fixed site was plotted in an “ancestry painting” if at least 80% of genotypes were complete using available ruby scripts (https://github.com/mmatschiner/tutorials/tree/master/analysis_of_introgression_with_snp_data, last accessed November 2021). These scripts calculate the heterozygosity of each individual and visualize regions that are possibly affected by introgression. The mitochondrial genome assembly from each individual was used to determine the maternal species and the proportion of the genome derived from the maternal species was then calculated for each hybrid. For gal*×*cuc hybrids the hybrid index scale ranges from 0 (gal) to 1 (cuc) and for gal*×*long hybrids from 0 (long) to 1 (gal).

#### Window-Based Population Parameters

To assess genome-wide genetic differentiation between the clusters identified with admixture, we calculated nucleotide diversity (*π*), between-taxon differentiation (*F*_ST_), and between-taxon divergence (*d*_xy_) using the Python script popgenWindows.py (github.com/simonhmartin/genomics_general release 0.3, Martin et al. 2020, last accessed November 2021) with a sliding 100-kb window, a step size of 10 kb and at least 20-kb genotyped sites within each window. To compare species pairs, we only considered individuals assigned to parental species based on ADMIXTURE results (supplementary table S2, Supplementary Material online). In addition, we also calculated these parameters using one randomly chosen individual from each population per species to check if the estimates are biased because of the overrepresentation of some populations in a species group (supplementary table S2, Supplementary Material online).

Sets of outlier windows were defined as those with *F*_ST_ values in the upper 95th percentile of the distribution for each of the three pairwise comparisons. Further, the genes in these windows were extracted using the annotation file. We used a randomization approach to assess whether the observed intersections (i.e., outlier *F*_ST_ windows occurring in both species) between all seven possible species comparisons are larger or smaller than expected by chance. For this, we randomly drew the observed number of windows, respectively genes from the total number of 10-kb windows in the assembly (13,330), respectively the total number of annotated genes (15,845) without replacement and calculated the intersections for all possible comparisons. We compared the resulting intersections from 1,000 replicates with the observed values (supplementary fig. S5, Supplementary Material online).

#### Inferring Introgression

To identify admixture among three populations, we calculated the *f*_3_ statistic with ADMIXTOOLS v 7.0 (Patterson et al. 2012) implemented in the admixr package in R (Petr et al. 2019). We used two parental source populations (A and B) and the target population (C) in the form (C; A, B). Significantly negative *f*_3_ statistic indicates that population C is a mixture of populations A and B or closely related populations.

#### Local Ancestry Inference

To prepare the SNP set, Beagle v4.1 was used to phase and impute genotypes with 10,000 bp step size and 1,000-bp overlapping sliding windows (Browning and Browning 2009). Local ancestry inference was conducted with Loter (Dias-Alves et al. 2018) which infers the origin of each SNP in an admixed individual from two ancestral source populations and does not require additional biological parameters. The respective two parental species populations were used to reconstruct the ancestry tracts of the three putative *galeata×cucullata* hybrid individuals and 11 putative *galeata×longispina* hybrid individuals using Loter with default settings. 

## Supplementary Material

Supplementary data are available at *Genome Biology and Evolution* online.

## Supplementary Material

evab267_Supplementary_Data
