# Supplementary material for: Hybridization Dynamics and Extensive Introgression in the Daphnia longispina Species Complex: New Insights from a High-Quality Daphnia galeata Reference Genome
Source: Genome Biol Evol. 2021 Dec 2;13(12):evab267. doi: 10.1093/gbe/evab267 (PMC8695838; doi:10.1093/gbe/evab267)
Supplement: evab267_Supplementary_Data [file evab267_supplementary_data.zip › GBE_SuppMethods.docx]

**Hybridization dynamics and extensive introgression in the *Daphnia longispina* species complex: new insights from a high-quality *Daphnia galeata* reference genome**

**Supplementary Materials and Methods**

In the following section default options are applied, if not stated otherwise.

**1. Genome assembly**

**Adapter and quality trimming**

Adapter and quality trimming of Illumina reads was conducted with autotrim.pl 0.6.1 (Waldvogel*, et al.* 2018), in combination with Trimmomatic 0.38 (Bolger*, et al.* 2014), FastQC 0.11.7 (Andrews 2010) and MultiQC 1.6 (Ewels*, et al.* 2016) using a custom adapter file and the following Trimmomatic parameters: ILLUMINACLIP: adapter_combined.fa:2:30:10 TRAILING:15 SLIDINGWINDOW:4:20 MINLEN:50.

The tool autotrim.pl allows to trim additionally to adapter and quality for overrepresented *k*-mers which probably arise from smaller parts of adapter sequences.

PacBio subreads were used as provided from the sequencing facility.

**Contamination screening on read level**

To filter out reads possibly originating from contamination, a FastQ Screen like (FQS-like) approach was chosen. In brief, the reads are separated according to mapping behavior to different genomes.

First, a database containing the genomes of *Daphnia magna* (Lee et al., 2019) and *D. pulex* (Ye*, et al.* 2017) as positive controls and the human genome, the genome of the algae the sequenced individuals were fed on as well as several bacterial and viral genomes as negative controls was created. The database contains 108,163 sequences with a total length of 42.4Gb (see Table 1). The accession numbers of the bacterial and viral genomes can be found in the corresponding provided lists.

Table 1: Database parts and corresponding sizes.

| **Species/group** | **Number of sequences** | **Total length [bp]** |
| --- | --- | --- |
| *D. magna* (dmagna-v2.4) | 40,356 | 131,266,987 |
| *D. pulex* (Daphnia_pulex_PA42_v3.0) | 1,822 | 156,418,198 |
| Bacteria | 18,448 | 37,434,584,693 |
| Human (GRCh38.p12) | 594 | 3,257,319,537 |
| *Acutodesmus* *obliquus* (GCA_002149895.1) | 2,707 | 208,176,092 |
| Virus | 44,236 | 1,170,153,228 |

Illumina reads were mapped unpaired (forward and reverse reads separately) with NextGenMap 0.5.5 (Sedlazeck*, et al.* 2013) and the options “‑‑bam 1 ‑‑bin_size 4 ‑‑topn 1000”. PacBio subreads were mapped with minimap2 2.17-r941 (Li 2018) and the options “-H -x map-pb”.

Custom scripts were used to filter reads and to display the results (<https://github.com/schellt/fqs-tools>). The mapping results to the different database parts are displayed in Figure 1. Reads did only pass the filtering if they either did not map to the database at all or had at least one hit against one of the two *Daphnia* genomes. Paired Illumina reads were only returned if both reads pass the filtering. If only one read of a pair passed the filtering they were returned as unpaired. An overview of the effect of different read filtering steps on data volume is shown in Table 2.

**Figure 1:** Mapping results


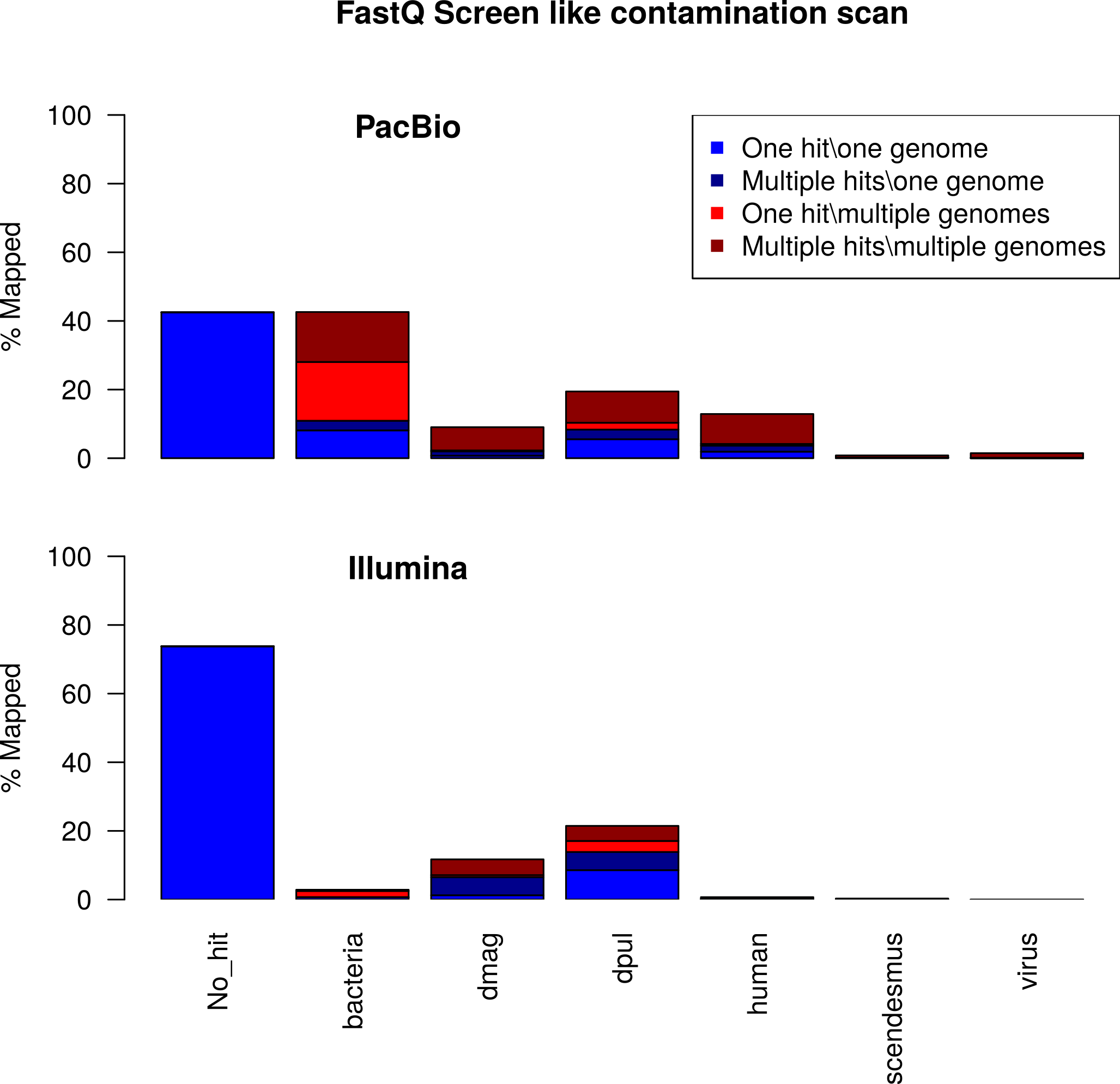


**Table 2:** Comparison of data amount regarding the different preprocessing steps.

|  | | **Raw** | | **Trimmed** | | **FQS-like filtered** | |
| --- | --- | --- | --- | --- | --- | --- | --- |
|  |  | #reads | Gb | #reads | Gb | #reads | Gb |
| Illumina | paired | 77,594,732 | 11.64 | 64,988,122 | 9.13 | 62,533,940 | 8.79 |
|  | unpaired | – | – | 5,322,216 | 0.66 | 5,962,964 | 0.76 |
| PacBio | | – | – | 1,679,290 | 11.52 | 1,121,227 | 6.84 |

**RA assembly**

All paired and unpaired contamination filtered Illumina reads as well as the contamination filtered PacBio reads were used as input for RA 0.2.1 (https://github.com/rvaser/ra).

**Contamination screening on assembly level**

To screen the resulting assembly blobtools 1.0 (Laetsch and Blaxter 2017) was used. The Illumina reads used as input for RA were mapped against the contigs using backmap.pl 0.1 (<https://github.com/schellt/backmap>), which integrates bwa mem 0.7.17-r1188 (Li 2013), samtools 1.9-33-g2d34e15 (Li*, et al.* 2009), Qualimap 2.2.1 (Okonechnikov*, et al.* 2016), bedtools 2.26.0 (Quinlan and Hall 2010) and R 3.5.0 (R Core Team, 2019). To assign Taxonomy IDs blastn 2.9.0+ (Camacho*, et al.* 2009) was used to align the contigs against the complete nt database (-task megablast -outfmt '6 qseqid staxids bitscore' -max_target_seqs 1 -max_hsps 1 -evalue 1e-25). For plotting blobtools 1.1.1 was used.

Contamination with different bacteria can be clearly seen in Figure 2. In total 267 contigs with coverage below 10x and/or GC content above 50% were removed. The removed contigs add up to a total length of 22.97Mb (see detailed Distribution in Figure 3).

To minimize false scaffolding later, PacBio reads mapping to the 267 contigs identified as contamination were removed. This resulted in 871,867 reads with a total length of 5.41Gb which corresponds to 78% and 79% of the FQS-like filtered reads respectively.


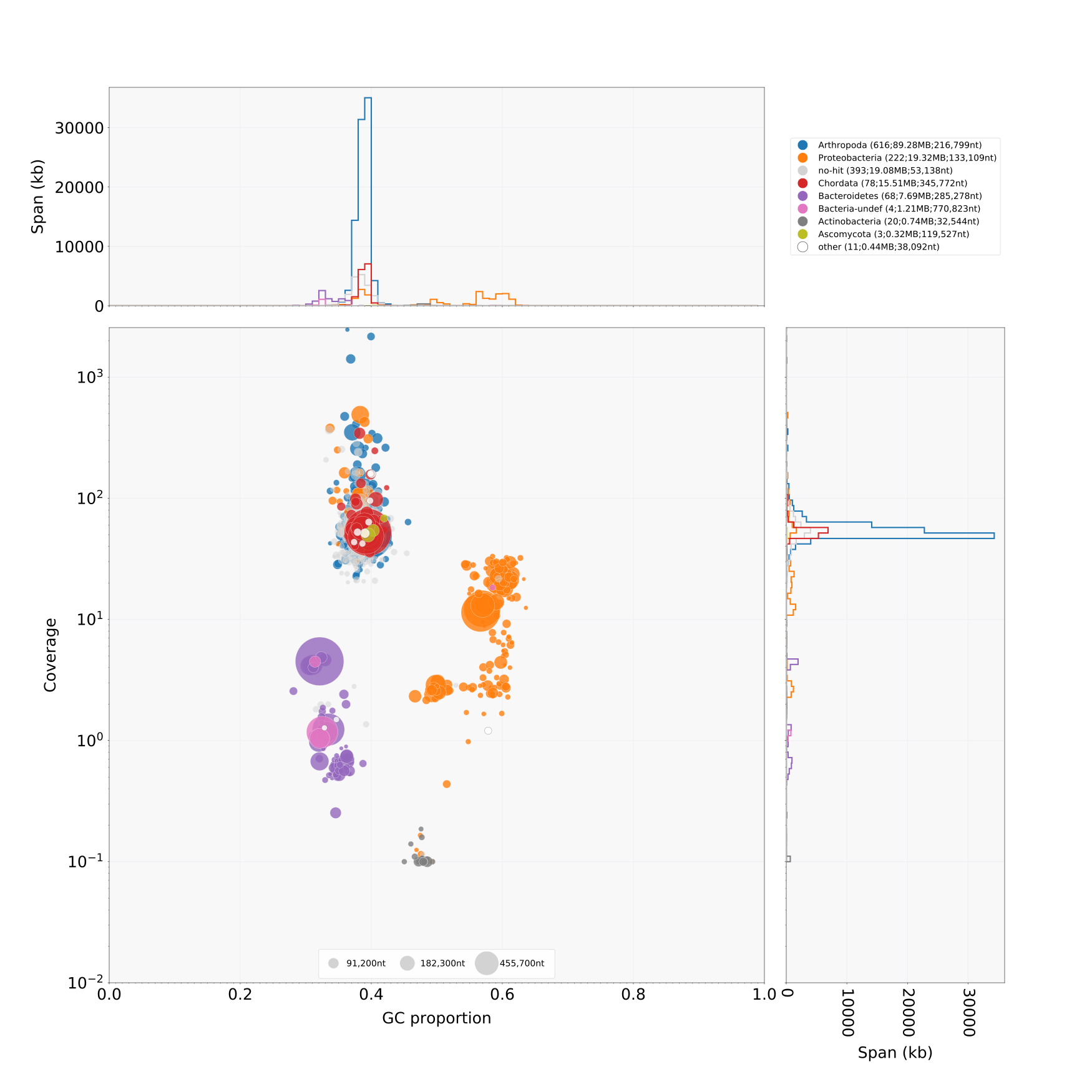

Figure 2: Blobplot of the inital RA assembly. Coverage is based on Illumina data only. Taxonomic assignment was conducted with blastn against the nt database.

**Identifying and removing the mitochondrial genome**

After assembly, the mitochondrial (mt) genome was searched with blastn 2.9.0+ by using the available *D. galeata* mt genomes (accession numbers LC177072.1, LC152879.1, LC177110.1, NC_034297.1, LC177071.1, LC177070.1) as queries against the contigs remaining after blobtools filtering. A total of 15 contigs had hits to at least one of the*se* mt genomes. However, one single contig could be identified by calculating the ratio between alignment length and target sequence (contig) length. The mt genome could be distinguished by the maximum ratio 0.98 from random hits which reached maximally a ratio of 0.008. Finally, one single contig with a length of 15,636bp was removed.

**Scaffolding and gap closing**

Scaffolding and gap closing was conducted in three iterations. Each iteration contains I) a scaffolding with SSPACE LongRead 1-1 (Boetzer and Pirovano 2014), II) gap closing with LR_Gapcloser (<https://github.com/CAFS-bioinformatics/LR_Gapcloser>; commit 156381a) and III) polishing of the former gap parts (that were filled with PacBio reads only) with short reads using wtdbg2-racon-pilon.pl 0.4 (<https://github.com/schellt/wtdbg2-racon-pilon>), which used bwa mem 0.7.17-r1188, samtools 1.9, java 1.8.0_221 (Arnold*, et al.* 2000) and Pilon 1.23 (Walker*, et al.* 2014).

The blobtools filtered PacBio reads were used for scaffolding and gapclosing. Coordinates of gaps produced in the scaffolding step were identified and written into bed format with the help of bedtools 2.28.0. Afterwards the FQS-like filtered paired and unpaired Illumina reads were mapped via bwa mem (-a -c 10000 -t 60) to the gap closed scaffolds. Only reads aligning at least partially to former gap regions were saved to bam format as well as sorted and indexed with samtools. Finally, Pilon was run with additional options “--‑diploid ‑--‑threads 60” on the produced bam files. After running Pilon the scaffold IDs were renamed to match the ones in the bed file again. Short read mapping and Pilon polishing was executed three times in iterative fashion using in all three polishing iterations the gap regions produced at the beginning from the long read‑ scaffolding iteration.

After the third iteration of scaffolding, gap closing and polishing the final scaffolds are yielded.

**Assembly quality assessment**

Contiguity was analyzed with Quast 5.0.2 (Gurevich*, et al.* 2013) at different stages of the assembly process and its main results represented in Table 3.

Table 3: Contiguity statistics of the single assembly steps.

|  | **ra** | **ra-blobfilter-rmmt** | **ra-blobfilter-scaff1** | **ra-blobfilter-scaff2** | **ra-blobfilter-scaff3** |
| --- | --- | --- | --- | --- | --- |
| #Sequences | 1,415 | 1,147 | 473 | 370 | 346 |
| Total length [Mb] | 153.6 | 130.6 | 132.9 | 133.2 | 133.3 |
| N50 [kb] | 172.4 | 175.3 | 533.0 | 729.4 | 756.7 |
| GC [%] | 40.02 | 38.74 | 38.75 | 38.75 | 38.75 |
| N’s [%] | ~0 | ~0 | 0.07 | 0.09 | 0.09 |

To look at mapping rate, coverage and insert size distribution, etc., backmap 0.3 (<https://github.com/schellt/backmap>) was used in combination with bwa mem 0.7.17-r1188, Minimap 2.17-r941, Samtools 1.9, Qualimap 2.2.1, bedtools 2.28.0, R 3.5.3 and MultiQC 1.8. Mapping the paired and unpaired FQS-like filtered Illumina reads and the blobtools filtered PacBio reads resulted in a mapping rate of 94.1% and 85.5%, respectively, according to Qualimap’s bamqc. The insert size of the paired Illumina reads is narrowly distributed around a median of 327 (Figure 3). The genome size was estimated based on mapped nucleotides and mode of the coverage distribution by backmap, resulting in 156.86Mb and 178.03Mb for Illumina (52x) and PacBio (26x) respectively. Additionally, the genome size estimated using a k-mer based approach with GenomeScope (150.6Mb) can be found here: <http://qb.cshl.edu/genomescope/analysis.php?code=WeYH4KGn4W7eTjIa1qhf>.

To show absence of contamination in the assembly a blastn search of the final scaffolds against the nt database was conducted as above, and the results plotted with blobtools 1.1.1.

Completeness in terms of single copy core orthologs of the final scaffolds was assessed with BUSCO 3.0.2 (Simão*, et al.* 2015), using the Arthropoda set (odb9) and the option --‑long. This resulted in C: 95.7% [S: 94.7%, D: 1.0%], F: 0.8%, M: 3.5%, n‑: 1066.


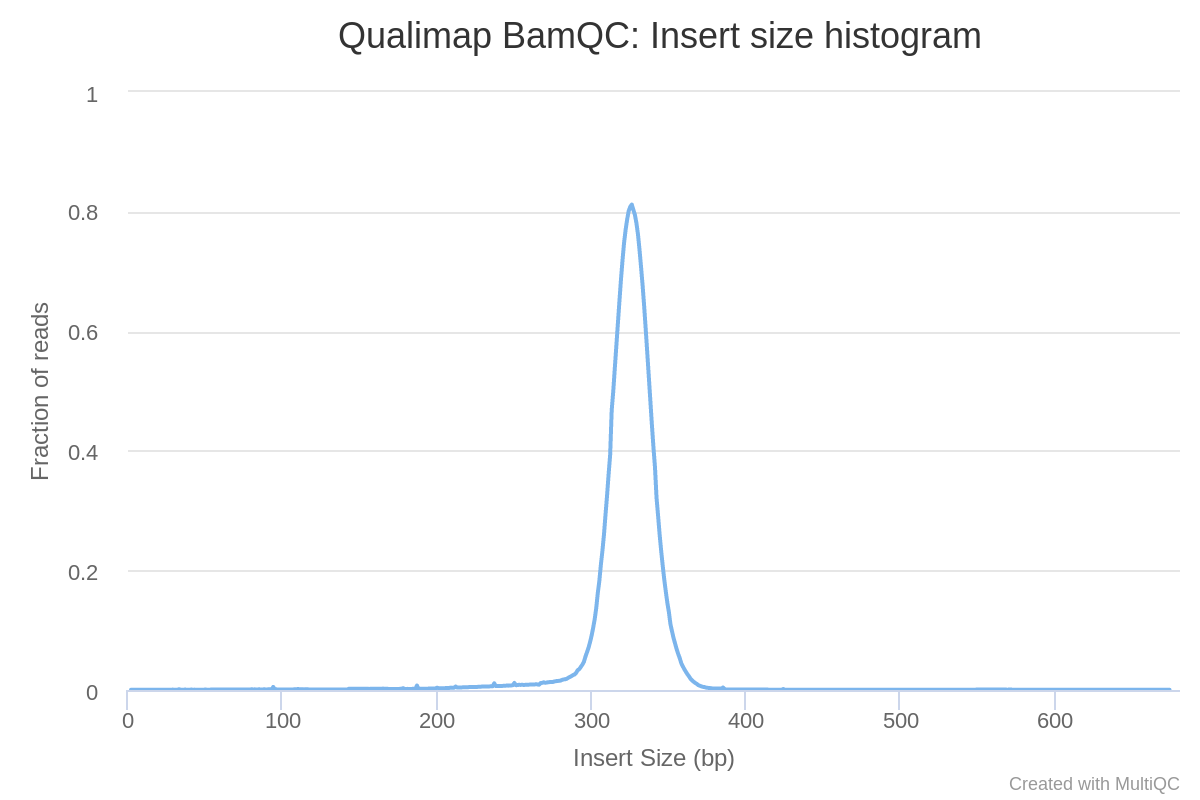


Figure 3: Insert size distribution of paired Illumina reads. Created with Qualimap and MultiQC.

**2. Genome annotation**

Before starting with the annotation the fasta headers of the genome assembly and the *D. galeata* transcriptome assembly (HAFN01.1) were simplified with Augustus’ simplifyFastaHeaders.pl.

**Repeat library creation and repeat masking**

To identify *D. galeata* specific repeats, RepeatModeler 2.0 (Smit and Hubley 2015) in combination with RepeatMasker 4.1.0 (Smit*, et al.* 2013-2015) including RepBase release 20181026 (Bao*, et al.* 2015), RECON 1.08 (Bao and Eddy 2002), RepeatScout 1.0.6 (Price*, et al.* 2005), Tandem Repeats Finder 4.0.9 (Benson 1999) and RMBlast 2.9.0+. RepeatModeler was run with the options “-pa 10 -LTRStruct” and resulted in 1,115 families with a total length of ~1Mb. Detailed distribution of repeat family classification can be seen in Table 4.

Table 4: Distribution of repeat family classification.

| **Classification** | **Number of families** | **Total length [bp]** |
| --- | --- | --- |
| Unknown | 796 | 474,907 |
| LTR | 145 | 249,242 |
| DNA | 93 | 155,424 |
| LINE | 35 | 70,649 |
| tRNA | 31 | 43,674 |
| SINE | 13 | 17,071 |
| RC | 5 | 7,080 |
| Simple repeat | 4 | 364 |
| SINE | 2 | 5,442 |
| snRNA | 1 | 3,215 |

The yielded repeat families were combined with 237 *D. pulex* and 1 *D. pulicaria* repeat sequences from RepBase release 20181026 to create the final repeat library with 1,353 sequences and a total length of 1.78Mb.

The genome assembly was then soft masked for later usage within Augustus with the final repeat library. To do so, RepeatMasker 4.1.0 was applied with the search engine rmblastn 2.9.0+, Tandem Repeats Finder 4.0.9 and the additional options “-xsmall -no_is -e ncbi -pa 10 -s”. Afterwards 21.9% of the assembly were masked. The distribution of masked fraction per repeat element can be found in Table 5.

Table 5: Repeat masking results as provided from RepeatMasker. Most repeats fragmented by insertions or deletions have been counted as one element.

| **Classification** | **Number of elements** | **Percentage of assembly** |
| --- | --- | --- |
| Unknown | 48,249 | 10.18 |
| LTR | 7,824 | 5.25 |
| DNA | 7,145 | 2.29 |
| Simple repeat | 60,548 | 1.58 |
| LINE | 2,631 | 1.36 |
| snRNA | 1,591 | 0.49 |
| Low complexity | 12,696 | 0.43 |
| SINE | 849 | 0.19 |
| RC | 444 | 0.13 |

**Creation of initial gene prediction models**

Three different gene prediction models were produced with Augustus, GeneMark and SNAP. The detailed procedure is described below.

One model was created using autoAug.pl from Augustus 3.3.2 (Stanke*, et al.* 2008) in combination with PASA 2.4.1 (Haas*, et al.* 2003) and GMAP 2019-09-12 (Wu and Watanabe 2005). As Input for autoAug.pl were used the soft masked assembly and the *D. galeata* transcriptome (HAFN01.1, Huylmans*, et al.* 2016) as well as the additional options “-v ‑-v ‑-v ‑--‑pasa ‑--‑useGMAPforPASA ‑--‑‑noninteractive”.

A second model was created using GeneMark ET 4.48_3.60_lic (Lomsadze*, et al.* 2005). First different RNAseq reads (ERR1551794 and ERR1551390- ERR1551397) were trimmed with autotrim.pl. The trimmed RNAseq reads where mapped paired and unpaired separately with HISAT 2.1.0 (Kim*, et al.* 2019) and the additional option “-p 70”. The two resulting bam files were merged and sorted (‑-l 9 ‑-@ 10) using samtools 1.9. Afterwards Augustus’ bam2hints with the additional options “‑--‑minintronlen=20 ‑--‑maxintronlen=500000” was used to produce a gff file containing possible introns. This gff file was filtered using filterIntronsFindStrand.pl (included in Augustus) with the additional option “‑--‑score”. Finally, gmes_petap.pl from GeneMark was run with the unmasked genome assembly, the filtered intron gff file and the additional options “‑--‑v ‑--‑cores=88 ‑--‑‑max_intron=400000”.

A third model was created with busco2snap.pl 0.1 (<https://github.com/schellt/busco2snap>) in combination with SNAP 2006-07-28 (Korf 2004), Augustus 3.3.2 and blastp 2.9.0+. In brief, the intron-exon-boundaries of 1010 complete and single copy BUSCOs are predicted with the Augustus model created with BUSCO. If more than one gene is predicted per locus a blastp search against the ancestral variants of the BUSCO genes is used to select the most likely one. Afterwards the exon annotation is converted from gff to zff and finally the SNAP model is created with its tools fathom, forge and hmm-assembler.pl.

**Structural annotation**

The structural annotation was conducted in MAKER 2.31.10 (Holt and Yandell 2011) in combination with blast 2.10.0+, RepeatMasker 4.1.0, Exonerate 2.4.0 (Slater and Birney 2005), SNAP 2013-11-29, GeneMark 3.60_lic, Augustus 3.3.2 and tRNAscan-SE 1.3.1 (Lowe and Eddy 1997). The following input sequences were used for MAKER: the unmasked genome assembly, the species own transcriptome assembly (HAFN01.1) as ESTs, the complete Swiss-Prot 2019_10 (Consortium 2019), and the protein sequences resulting from *D. magna* (Lee*, et al.* 2019) as well as *D. pulex* (Ye*, et al.* 2017) genome annotations as protein evidence. Repeat masking was conducted with the above described final repeat library for RepeatMasker and MAKER’s te_proteins.fasta for RepeatRunner. For model based gene prediction the above described models from SNAP, GeneMark and Augustus were fed into MAKER. The options est2genome, protein2genome, trna and alt_splice were switched on. The minimum protein length was set to 10aa. MAKER was compiled and executed with mpich 3.3.2 and the additional option “-fix_nucleotides”. With this, the first round of MAKER was completed.

Afterwards one gff file with and one without the assembly sequence were created using MAKER’s gff3_merge. From the gff without assembly sequence the tracks “est2genome”, “protein2genome” and “repeatmasker”/”repeatrunner” were extracted and saved in three separate gff files.

The gff file with assembly sequence was used as input for MAKER’s maker2zff. The resulting files were processed with fathom (fathom genome.ann genome.dna -categorize 1000 && fathom -export 1000 -plus uni.ann uni.dna), forge (forge export.ann export.dna) and hmm-assembler.pl to create a new SNAP model based on the first round of MAKER.

The Augustus model was retrained with autoAug.pl as above, except that the gff file without assembly sequence from first round’s results was specified as trainingset.

The second round of MAKER was conducted as the first round, but instead of specifying the same evidence and repeat sequences again, the gff files containing the tracks est2genome, protein2genome and repeats as est_gff, protein_gff and rm_gff were used respectively. Furthermore, the retrained/new models from Augustus and SNAP were used.

After finishing the second round of MAKER, the Augustus and SNAP model were retrained/newly created as above.

The third and final round of MAKER was run as the second one, except of updating the Augustus and SNAP model. The options est2genome and protein2genome were switched off, whereas the option always_complete was switched on.

Finally, a gff file containing the maker track only without the assembly sequence was created with gff3_merge. The transcripts and protein fasta files of the annotated genes were created with fasta_merge.

**Structural annotation quality assessment**

The final gff file was processed with a custom script to count and calculate several contiguity statistics of the structural annotation except tRNAscan results. Furthermore, the annotated protein set was analyzed using BUSCO 3.0.2 in combination with the Arthropoda set (odb9) and DOGMA 3.4 (Dohmen*, et al.* 2016) in combination with Pfam scan 1.6 and DOGMA’s reference set for Arthropoda. The results are compared in Table 6.

Table 6: Structural annotation statistics. Contiguity statistics of the annotation were calculated excluding tRNAscan results. BUSCO 3.0.2 was executed in protein mode for the different MAKER rounds. Conserved Domain Arrangements (CDAs) were searched with Pfam scan 1.6 and DOGMA 3.4.

|  | **Assembly** | **Round 1** | **Round 2** | **Round 3** |
| --- | --- | --- | --- | --- |
| Number |  |  |  |  |
| Gene |  | 15513 | 15429 | 15845 |
| mRNA |  | 16471 | 16275 | 16774 |
| Exon |  | 121995 | 119033 | 117364 |
| CDS |  | 130242 | 123265 | 119402 |
| Mean number of |  |  |  |  |
| mRNAs/gene |  | 1.06 | 1.05 | 1.06 |
| Exons/mRNA |  | 7.41 | 7.31 | 7.00 |
| CDSs/mRNA |  | 7.91 | 7.57 | 7.12 |
| Median length |  |  |  |  |
| Gene |  | 2145 | 2187 | 2097 |
| mRNA |  | 2236 | 2248 | 2142 |
| Exon |  | 163 | 164 | 167 |
| Intron |  | 74 | 74 | 74 |
| CDS |  | 150 | 151 | 152 |
| Total space |  |  |  |  |
| Gene |  | 54765381 | 52605643 | 51689473 |
| mRNA |  | 54765288 | 52605588 | 51689329 |
| Exon |  | 29662778 | 29222288 | 29314592 |
| CDS |  | 25750445 | 25271022 | 25132876 |
| Single |  |  |  |  |
| Exon mRNA |  | 1015 | 746 | 663 |
| CDS mRNA |  | 1104 | 854 | 710 |
|  |  |  |  |  |
| BUSCO [%] N=1066 |  |  |  |  |
| Complete | 95,7 | 93,9 | 93,9 | 94,3 |
| Single copy | 94,7 | 91,0 | 91,0 | 91,7 |
| Duplicated | 1,0 | 2,9 | 2,9 | 2,6 |
| Fragmented | 0,8 | 0,8 | 1,0 | 0,7 |
| Missing | 3,5 | 5,3 | 5,1 | 5,0 |
| CDAs [%] N=4222 |  | 93,94 | 93,89 | 93,63 |

As further quality criterion the Annotation Editing Distance (AED) was compared between the three MAKER rounds and is visualized in Figure 4.


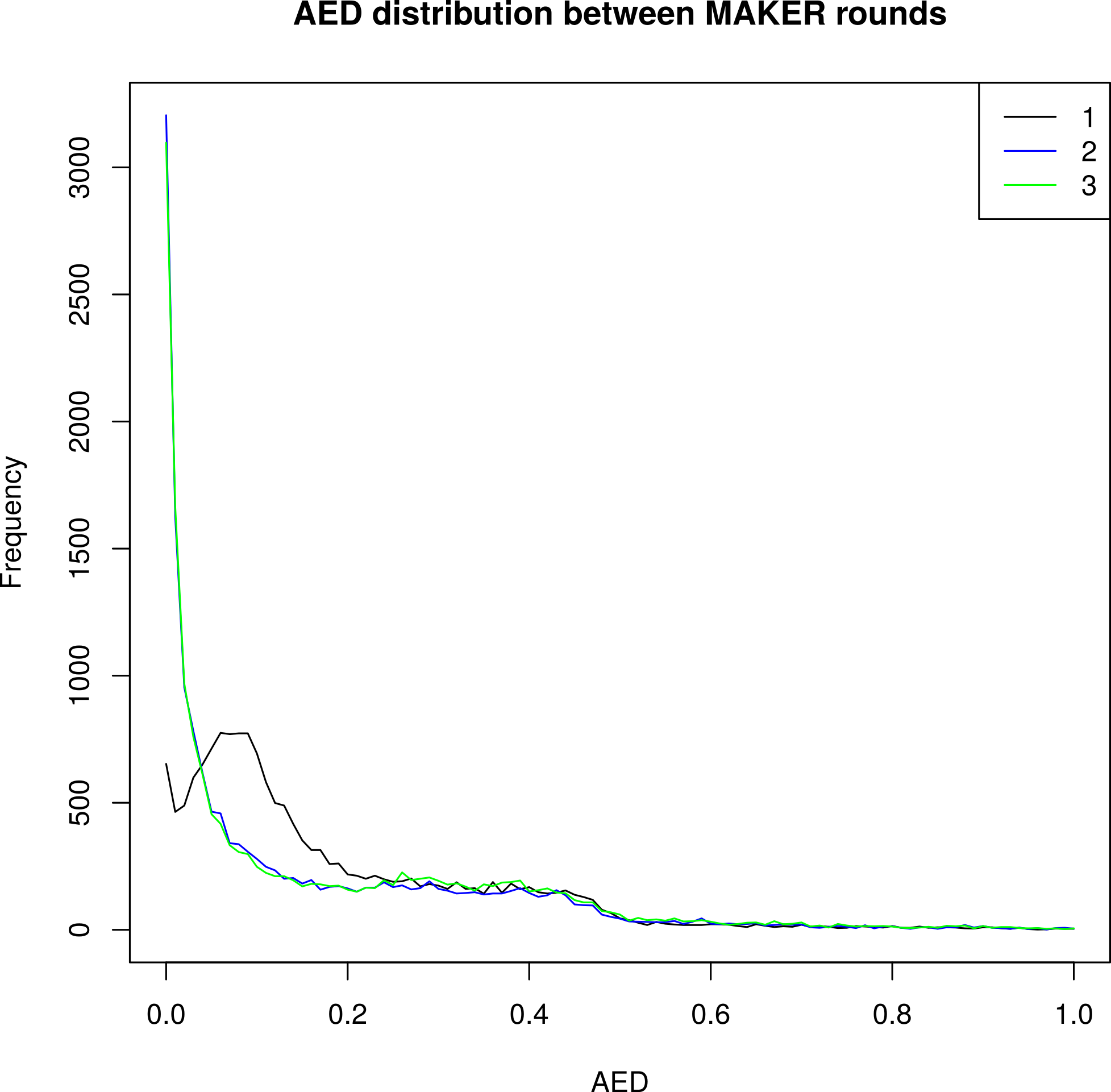

Figure 4: Annotation Editing Distance (AED) distribution of the different MAKER iterations. The AED shows congruency between the annotation and evidence alignments. Low values indicate high congruency.

**Functional annotation**

The functional annotation of the annotated protein set was conducted using InterProScan 5.39-77.0 (Jones*, et al.* 2014) in combination with the databases/analyses CDD 3.17 (Lu*, et al.* 2020), Coils 2.2.1 (Lupas*, et al.* 1991), Gene3D 4.2.0 (Yeats*, et al.* 2006), Hamap 2019_01 (Pedruzzi*, et al.* 2015), MobiDBLite 2.0 (Necci*, et al.* 2017), PANTHER 14.1 (Mi*, et al.* 2012), Pfam 32.0 (El-Gebali*, et al.* 2019), Phobius 1.01 (Käll*, et al.* 2004), PIRSF 3.02 (Wu*, et al.* 2004), PRINTS 42.0 (Attwood*, et al.* 2012), ProSitePatterns 2019_01 and ProSiteProfiles 2019_01 (Sigrist*, et al.* 2012), SFLD 4 (Akiva*, et al.* 2014), SignalP_EUK 4.1 (Nielsen 2017), SMART 7.1 (Letunic and Bork 2018), SUPERFAMILY 1.75 (Wilson*, et al.* 2009), TIGRFAM 15.0 (Haft*, et al.* 2001) and TMHMM 2.0c (Krogh*, et al.* 2001). The lookup for pathways and Gene Ontology (GO) terms was switched on.

To additionally assign putative names, an alignment of the annotated protein set against the Swiss-Prot 2019_10 was conducted using blastp 2.9.0+ with the options “‑num_threads 70 ‑max_hsps 1 ‑max_target_seqs 1 ‑outfmt 6”

.

In total 15,898 (94.78%) and 15,960 (95.15%) protein sequences could be annotated by InterProScan and blast against Swiss-Prot, respectively. In combination, a functional annotation for 16,675 (99.41%) protein sequences could be achieved. A detailed overview of the functional annotated sequences per database or search algorithm is shown in Table 7.

Table 7: Functional annotation success of the different Databases and search algorithms.

| **Database/analysis** | **Number of different protein sequences** |
| --- | --- |
| **Interpro** |  |
| CDD | 5550 |
| Coils | 3191 |
| Gene3D | 9977 |
| Hamap | 279 |
| MobiDBLite | 6661 |
| PANTHER | 13040 |
| Pfam | 11567 |
| Phobius | 6876 |
| PIRSF | 793 |
| PRINTS | 2565 |
| ProSitePatterns | 3169 |
| ProSiteProfiles | 6378 |
| SFLD | 84 |
| SignalP_EUK | 2951 |
| SMART | 5444 |
| SUPERFAMILY | 9816 |
| TIGRFAM | 721 |
| TMHMM | 4211 |
| GO | 9555 |
| Reactome | 3927 |
| **Total** | **15898** |
| **Total [%]** | **94.78** |
|  |  |
| **blast** |  |
| Swiss-Prot | 15960 |
| Swiss-Prot [%] | 95.15 |
|  |  |
| **Overall total** | **16737** |
| **Overall total [%]** | **99.78** |

The results of the functional annotation was then included in the gff and the transcript/protein fasta files using adapted versions of MAKER’s maker_functional_gff and maker_functional_fasta respectively (https://github.com/schellt/maker-functional).

**References**

Akiva E*, et al.* 2014. The structure–function linkage database. The structure–function linkage database 42: D521-D530.

Andrews S. 2010. FastQC: A quality control tool for high throughput sequence data.

Arnold K, Gosling J, Holmes D, Holmes D. 2000. The Java programming language: Addison-wesley Reading.

Attwood TK*, et al.* 2012. The PRINTS database: a fine-grained protein sequence annotation and analysis resource—its status in 2012. The PRINTS database: a fine-grained protein sequence annotation and analysis resource—its status in 2012 2012.

Bao W, Kojima KK, Kohany O. 2015. Repbase Update, a database of repetitive elements in eukaryotic genomes. Repbase Update, a database of repetitive elements in eukaryotic genomes 6: 11

Bao Z, Eddy SR 2002. Automated de novo identification of repeat sequence families in sequenced genomes. Automated de novo identification of repeat sequence families in sequenced genomes 12: 1269-1276.

Benson G 1999. Tandem repeats finder: a program to analyze DNA sequences. Tandem repeats finder: a program to analyze DNA sequences 27: 573-580.

Boetzer M, Pirovano W 2014. SSPACE-LongRead: scaffolding bacterial draft genomes using long read sequence information. BMC Bioinform. 15: 211.

Bolger AM, Lohse M, Usadel B 2014. Trimmomatic: a flexible trimmer for Illumina sequence data. Bioinformatics 30: 2114-2120. doi: 10.1093/bioinformatics/btu170

Camacho C*, et al.* 2009. BLAST+: architecture and applications. BMC Bioinform. 10: 421.

Consortium U 2019. UniProt: a worldwide hub of protein knowledge. UniProt: a worldwide hub of protein knowledge 47: D506-D515.

Dohmen E, Kremer LP, Bornberg-Bauer E, Kemena C 2016. DOGMA: domain-based transcriptome and proteome quality assessment. Bioinformatics 32: 2577-2581.

El-Gebali S*, et al.* 2019. The Pfam protein families database in 2019. The Pfam protein families database in 2019 47: D427-D432.

Ewels P, Magnusson M, Lundin S, Käller M 2016. MultiQC: summarize analysis results for multiple tools and samples in a single report. Bioinformatics 32: 3047-3048.

Gurevich A, Saveliev V, Vyahhi N, Tesler G 2013. QUAST: quality assessment tool for genome assemblies. Bioinformatics 29: 1072-1075.

Haas BJ*, et al.* 2003. Improving the *Arabidopsis* genome annotation using maximal transcript alignment assemblies. Improving the *Arabidopsis* genome annotation using maximal transcript alignment assemblies 31: 5654-5666.

Haft DH*, et al.* 2001. TIGRFAMs: a protein family resource for the functional identification of proteins. TIGRFAMs: a protein family resource for the functional identification of proteins 29: 41-43.

Holt C, Yandell M. 2011. MAKER2: an annotation pipeline and genome-database management tool for second-generation genome projects. BMC Bioinform. 12: 491

Huylmans AK, López Ezquerra A, Parsch J, Cordellier M 2016. De Novo Transcriptome Assembly and Sex-Biased Gene Expression in the Cyclical Parthenogenetic *Daphnia galeata*. Genome Biol. Evol 8: 3120-3139. doi: 10.1093/gbe/evw221

Jones P*, et al.* 2014. InterProScan 5: genome-scale protein function classification. Bioinformatics 30: 1236-1240.

Käll L, Krogh A, Sonnhammer EL 2004. A combined transmembrane topology and signal peptide prediction method. A combined transmembrane topology and signal peptide prediction method 338: 1027-1036.

Kim D, Paggi JM, Park C, Bennett C, Salzberg SL 2019. Graph-based genome alignment and genotyping with HISAT2 and HISAT-genotype. Nat. Biotechnol. 37: 907-915.

Korf I. 2004. Gene finding in novel genomes. BMC Bioinform. 5: 59

Krogh A, Larsson B, Von Heijne G, Sonnhammer EL 2001. Predicting transmembrane protein topology with a hidden Markov model: application to complete genomes. Predicting transmembrane protein topology with a hidden Markov model: application to complete genomes 305: 567-580.

Laetsch DR, Blaxter ML. 2017. BlobTools: Interrogation of genome assemblies. F1000Research: 6:1287

Lee B-Y*, et al.* 2019. The genome of the freshwater water flea *Daphnia magna*: A potential use for freshwater molecular ecotoxicology. Aquat. Toxicol. 210: 69-84.

Letunic I, Bork P 2018. 20 years of the SMART protein domain annotation resource. 20 years of the SMART protein domain annotation resource 46: D493-D496.

Li H. 2013. Aligning sequence reads, clone sequences and assembly contigs with BWA-MEM. arXiv: 1303.3997

Li H 2018. Minimap2: pairwise alignment for nucleotide sequences. Bioinformatics 34: 3094-3100.

Li H*, et al.* 2009. The Sequence Alignment/Map format and SAMtools. The Sequence Alignment/Map format and SAMtools 25: 2078-2079. doi: 10.1093/bioinformatics/btp352

Lomsadze A, Ter-Hovhannisyan V, Chernoff YO, Borodovsky M 2005. Gene identification in novel eukaryotic genomes by self-training algorithm. Nucleic Acids Res 33: 6494-6506.

Lowe TM, Eddy SR 1997. tRNAscan-SE: a program for improved detection of transfer RNA genes in genomic sequence. tRNAscan-SE: a program for improved detection of transfer RNA genes in genomic sequence 25: 955-964.

Lu S*, et al.* 2020. CDD/SPARCLE: the conserved domain database in 2020. CDD/SPARCLE: the conserved domain database in 2020 48: D265-D268.

Lupas A, Van Dyke M, Stock J 1991. Predicting coiled coils from protein sequences. Predicting coiled coils from protein sequences: 1162-1164.

Mi H, Muruganujan A, Thomas PD 2012. PANTHER in 2013: modeling the evolution of gene function, and other gene attributes, in the context of phylogenetic trees. PANTHER in 2013: modeling the evolution of gene function, and other gene attributes, in the context of phylogenetic trees 41: D377-D386.

Necci M, Piovesan D, Dosztányi Z, Tosatto SC 2017. MobiDB-lite: fast and highly specific consensus prediction of intrinsic disorder in proteins. MobiDB-lite: fast and highly specific consensus prediction of intrinsic disorder in proteins 33: 1402-1404.

Nielsen H. 2017. Predicting secretory proteins with SignalP. In. Protein function prediction: Springer. p. 59-73.

Okonechnikov K, Conesa A, García-Alcalde F 2016. Qualimap 2: advanced multi-sample quality control for high-throughput sequencing data. Qualimap 2: advanced multi-sample quality control for high-throughput sequencing data 32: 292-294.

Pedruzzi I*, et al.* 2015. HAMAP in 2015: updates to the protein family classification and annotation system. HAMAP in 2015: updates to the protein family classification and annotation system 43: D1064-D1070.

Price AL, Jones NC, Pevzner PA 2005. De novo identification of repeat families in large genomes. De novo identification of repeat families in large genomes 21: i351-i358.

Quinlan AR, Hall IM 2010. BEDTools: a flexible suite of utilities for comparing genomic features. BEDTools: a flexible suite of utilities for comparing genomic features 26: 841-842.

Sedlazeck FJ, Rescheneder P, Von Haeseler A 2013. NextGenMap: fast and accurate read mapping in highly polymorphic genomes. Bioinformatics 29: 2790-2791.

Sigrist CJ*, et al.* 2012. New and continuing developments at PROSITE. New and continuing developments at PROSITE 41: D344-D347.

Simão FA, Waterhouse RM, Ioannidis P, Kriventseva EV, Zdobnov EM 2015. BUSCO: assessing genome assembly and annotation completeness with single-copy orthologs. Bioinformatics 31: 3210-3212. doi: 10.1093/bioinformatics/btv351

Slater GSC, Birney E. 2005. Automated generation of heuristics for biological sequence comparison. Automated generation of heuristics for biological sequence comparison 6: 31

Smit AFA, Hubley R. 2015. RepeatModeler Open. Version 1.0.

Smit AFA, Hubley R, Green P. 2013-2015. RepeatMasker Open-4.0.

Stanke M, Diekhans M, Baertsch R, Haussler D 2008. Using native and syntenically mapped cDNA alignments to improve de novo gene finding. Bioinformatics 24: 637-644.

Waldvogel AM*, et al.* 2018. The genomic footprint of climate adaptation in *Chironomus riparius*. Mol. Ecol. 27: 1439-1456.

Walker BJ*, et al.* 2014. Pilon: an integrated tool for comprehensive microbial variant detection and genome assembly improvement. PLoS ONE 9: e112963

Wilson D*, et al.* 2009. SUPERFAMILY—sophisticated comparative genomics, data mining, visualization and phylogeny. SUPERFAMILY—sophisticated comparative genomics, data mining, visualization and phylogeny 37: D380-D386.

Wu CH*, et al.* 2004. PIRSF: family classification system at the Protein Information Resource. PIRSF: family classification system at the Protein Information Resource 32: D112-D114.

Wu TD, Watanabe CK 2005. GMAP: a genomic mapping and alignment program for mRNA and EST sequences. GMAP: a genomic mapping and alignment program for mRNA and EST sequences 21: 1859-1875.

Ye ZQ*, et al.* 2017. A New Reference Genome Assembly for the Microcrustacean *Daphnia pulex*. G3 7: 1405-1416. doi: 10.1534/g3.116.038638

Yeats C*, et al.* 2006. Gene3D: modelling protein structure, function and evolution. Gene3D: modelling protein structure, function and evolution 34: D281-D284.
